# Light and Temperature Signalling at the Level of *CBF14* Gene Expression in Wheat and Barley

**DOI:** 10.1007/s11105-017-1035-1

**Published:** 2017-05-12

**Authors:** Aliz Novák, Ákos Boldizsár, Krisztián Gierczik, Attila Vágújfalvi, Éva Ádám, László Kozma-Bognár, Gábor Galiba

**Affiliations:** 1 Agricultural Institute, Centre for Agricultural Research, Hungarian Academy of Sciences, Martonvásár, Hungary; 2 Festetics Doctoral School, Georgikon Faculty, University of Pannonia, Keszthely, Hungary; 3 Biological Research Centre, Hungarian Academy of Sciences, Szeged, Hungary; 4 Department of Genetics, Faculty of Sciences and Informatics, University of Szeged, Szeged, Hungary

**Keywords:** Cereals, CBF14, Monochromatic light, Phytochrome, Cryptochrome

## Abstract

The wheat and barley *CBF14* genes have been newly defined as key components of the light quality-dependent regulation of the freezing tolerance by the integration of phytochrome-mediated light and temperature signals. To further investigate the wavelength dependence of light-induced *CBF14* expression in cereals, we carried out a detailed study using monochromatic light treatments at an inductive and a non-inductive temperature. Transcript levels of *CBF14* gene in winter wheat Cheyenne, winter einkorn G3116 and winter barley Nure genotypes were monitored. We demonstrated that (1) *CBF14* is most effectively induced by blue light and (2) provide evidence that this induction does not arise from light-controlled *CRY* gene expression. (3) We demonstrate that temperature shifts induce *CBF14* transcription independent of the light conditions and that (4) the effect of temperature and light treatments are additive. Based on these data, it can be assumed that temperature and light signals are relayed to the level of *CBF14* expression via separate signalling routes.

**Electronic supplementary material:**

The online version of this article (doi:10.1007/s11105-017-1035-1) contains supplementary material, which is available to authorized users.

## Introduction

Plants have developed adaptive mechanisms to integrate different environmental signals. The proper integration of the two most important external factors—light and temperature—is vital for proper development and acclimatization (Franklin 2009). In natural environments, light and temperature often change in parallel. Plants are able to distinguish differences of 1 °C, but the mechanisms of temperature perception have been largely unknown until now. Two recent publications revealed that phyB plays role as a temperature sensor in Arabidopsis (Jung et al. 2016; Legris et al. 2016). The mechanism behind this function is the temperature-dependent dark reversion (relaxation of the active Pfr form to the inactive Pr form) of the phyB photoreceptor.

It is well known that light signals drive photomorphogenic development of plants, but light is also considered as a modulator of responses to certain abiotic stress conditions, such as cold stress (Franklin 2009; Franklin et al. 2014). Significant amount of data on the interaction of light signal transduction and freezing tolerance has been accumulated in the recent years (Kim et al. 2002; Catala et al. 2011; Majláth et al. 2012; Maibam et al. 2013). In Arabidopsis, the crosstalk of these two processes occurs through a few key components, including the light sensing photoreceptors and members of the CBF/DREB (C-repeat binding factor/dehydration element binding factor) transcription factor family (Franklin and Whitelam 2007; Thomashow 2010; Mizoi et al. 2012). These transcription factors up-regulate the expression of cold responsive (COR) genes, resulting in increased freezing tolerance (Jaglo-Ottosen et al. 1998).

The Arabidopsis phytochrome family counts five members (phyA-E) (Sharrock and Quail 1989; Clack et al. 1994), but the monocot family contains only the light labile phyA and the light stabile phyB and phyC (Dehesh et al. 1991; Mathews and Sharrock 1997; Basu et al. 2000; Szűcs et al. 2006). They are red/far-red (R/FR) light-sensing photoreceptors and function as photoreversible light switches and are activated and inactivated upon perception of R and FR light, respectively.

Cryptochromes (CRY) are flavoproteins and show similarity to photolyases, but they do not have DNA repair activity (Todo 1999). They are mainly blue (B) and UV-A receptors and have important roles in photomorphogenesis. In Arabidopsis, two CRY proteins (CRY1 and CRY2) have been identified, differing in their C-terminal extension (Lin and Shalitin 2003). In wheat and barley three members of the cryptochrome family exist, CRY1a, CRY1b and CRY2 (Szűcs et al. 2006; Xu et al. 2009). The nomenclature of photoreceptors is based on protein sequence homology and does not necessarily reflect strict functional similarity between the dicot and monocot counterparts.

CBFs are members of the APETALA2 (AP2)/ethylene-responsive element binding protein transcription factor (TF) family. These types of TFs carry the AP2 DNA binding domain that interacts with C-repeat element(s) in the promoter of their target genes (Jaglo et al. 2001), thus regulating abiotic stress responses, mainly the cold response.

Many *CBF* genes are regulated by cold temperatures (Campoli et al. 2009), light quality (Franklin and Whitelam 2007; Novák et al. 2016), day length (Lee and Thomashow 2012) and the circadian clock. The interaction of phytochromes and the CBF pathway was first studied in Arabidopsis (Franklin and Whitelam 2007). Low R/FR ratio, which occurs at dusk or dawn, combined with lower temperature prepares the plant for the sudden drop of temperature in the night. Those plants, which were grown under low R/FR ratio, showed enhanced *CBF* expression and were more frost tolerant than their peers grown under normal white light (Franklin and Whitelam 2007). Low R/FR ratio partially inactivates the phyB receptor, which enables the accumulation of phytochrome-interacting factors (PIFs). Phytochrome-Interacting Factor 7 (PIF7) was shown to negatively regulate the expression of *DREB1C* (*CBF2*) transcripts. PIF7 activity was controlled by TOC1, a component of the circadian oscillator, as well as by phyB (Kidokoro et al. 2009). The antagonistic role of phyA and phyB has recently been described. In tomato plants, increased *CBF* transcript levels and freezing tolerance were observed under low R/FR ratio in *phyB* mutant and wild-type plants, but not in phyA-deficient mutants suggesting the positive role for phyA in the regulation of *CBF* genes (Wang et al. 2016). Light also mediates freezing tolerance through a newly identified CBF-independent pathway governed by the ELONGATED HYPOCOTYL 5 (HY5) TF. (Catala et al. 2011).

In cereals, limited information is available on the interaction of light and freezing tolerance and the components are not as well characterized as in Arabidopsis, despite the obvious agricultural implications of this phenomenon. This is mostly due to the lack of mutant collections that could facilitate the characterization of the signal transduction components, as it was done in Arabidopsis. However, there are a few reports starting to uncover the crosstalk of light and temperature signalling in cereals. According to Crosatti et al. (Crosatti et al. 1999), the accumulation of the barley COR14b protein (one of the targets of CBFs) is induced by R and B, but not by FR light. Vashegyi et al. (Vashegyi et al. 2013) examined the cold induced *CBF9, CBF14* and *COR14b* expression in light grown barley seedlings and dark grown barley callus and found that the induction is independent of the photosynthesis.

The high level of the CBF14 TF in wheat and barley is important for winter survival. Overexpressing wheat *TaCBF14* at a non-acclimating temperature caused increased freezing tolerance in transgenic spring barley plants (Soltész et al. 2013). Expression analysis of *CBF14* indicated that this gene is expressed at higher levels in winter wheat than in spring wheat and winter cultivars dispose higher copy number of *CBF14* than spring cultivars (Francia et al. 2007; Dhillon and Stockinger 2013; Galiba et al. 2013).

The light-quality regulation of *CBF14* was studied in wheat and barley by Novák et al. (2016). Supplementary FR light added to white light (low R/FR ratio) increased *CBF14* expression and freezing tolerance at a non-acclimating temperature (15 °C) (Novák et al. 2016). This response was attributed to the phytochrome system. The negative influence of phyB and the positive influence of phyA on the *CBF14* gene expression have been described in wheat, but not in einkorn, on a genotype-dependent manner (Novák et al. 2016). In contrast, the effect of CRY-mediated B light signals on the expression of *CBF* genes has not been reported yet.

To further investigate the wavelength dependence of light-induced *CBF14* expression in cereals, we carried out a set of experiments using monochromatic light treatments. R, FR and B light irradiation—absorbed by different photoreceptors—was applied at an inductive and a non-inductive temperature and transcript levels of the wheat and barley *CBF14* genes were monitored. Here, we show that *CBF14* is most effectively induced by B light and provide evidence that this induction does not arise from light-controlled *CRY* gene expression. We demonstrate that temperature shifts induce *CBF14* transcription independent of the light conditions and that the effect of temperature and light treatments are additive. Thus, it can be assumed that temperature and light signals are relayed to the level of *CBF14* expression via separate signalling routes.

## Materials and Methods

### Plant Materials and Growth Conditions

The winter genotype of barley (*Hordeum vulgare* subsp*. vulgare*) cultivar Nure, the winter wheat *Triticum aestivum* cv. ‘Cheyenne’ and the winter einkorn *Triticum monococcum* ‘G3116’ were used in this study. Plantlets were established in 44 mm Jiffy-7 peat rooting media (Jiffy International, Kristiansand, Norway) and grown in a Conviron PGR-15 growth chamber set at continuous 20 °C day/night temperature, 12-h day length and 250 μmol m^−2^ s^−1^ light intensity for 2 weeks.

### Treatments with Monochromatic Lights

Two-week-old plantlets were dark adapted for 2 days at 20 °C and then treated with white or different monochromatic lights for 4 or 8 h at 20 or 15 °C using B (450 nm), R (660 nm) or FR (735 nm) LED panels producing 500 μW/cm^2^. White light was produced by Tungsram HgMIF 400 W/DH metal halide light sources at 1200 μW/cm^2^ intensity. Control plants were maintained in the dark. Leaf samples from three plantlets were collected after 4 and 8 h of light or dark treatment, immediately frozen in liquid N_2_ and stored at −80 °C until analysis.

### Gene Expression Analysis

Total RNA was extracted from leaf samples stored at −80 °C using the Direct-zol™ RNA MiniPrep kit (Zymo Research, Irvine, CA, USA) and quantified by Nanodrop 1000 (Thermo Scientific, Wilmington, DE, USA). Synthesis of cDNA was done from 1 μg of total RNA using M-MLV Reverse Transcriptase (Affymetrix, Santa Clara, CA, USA) according to supplier’s protocol. The KAPA SYBR^®^ FAST Universal 2× qPCR Master Mix (Kapabiosystems, Wilmington, USA), gene specific and house-keeping primers (**S1** Table; (Burton et al. 2004; Paolacci et al. 2009; Campoli et al. 2009; Dhillon et al. 2010; Morran et al. 2011; Boldizsár et al. 2016; Novák et al. 2016)) and CFX96 Touch™ Real-Time PCR Detection System (Bio-Rad, Hercules, CA, USA) were used for quantitative real-time PCR reactions. The relative gene expressions were calculated using the ΔΔCt method (Livak and Schmittgen 2001), where Ct values were normalized by the Ct values of house-keeping genes (*cyclophilin* for barley and the *Ta30797 phosphogluconate dehydrogenase* for wheat) and relative to the control samples.

### Statistical Analysis

For the statistical analyses, one-way ANOVA and a least significant difference test, or Tukey’s *b* post hoc test or a Mann–Whitney non-parametric test (if any condition was not fulfilled) was performed using SPSS 16.0. The normality was tested by a Kolmogorov–Smirnov probe, and the homogeneity of the variances was tested by Levene’s test.

## Results

### Temperature- and Light-Dependent Induction of *CBF14*

**Fig. 1:**
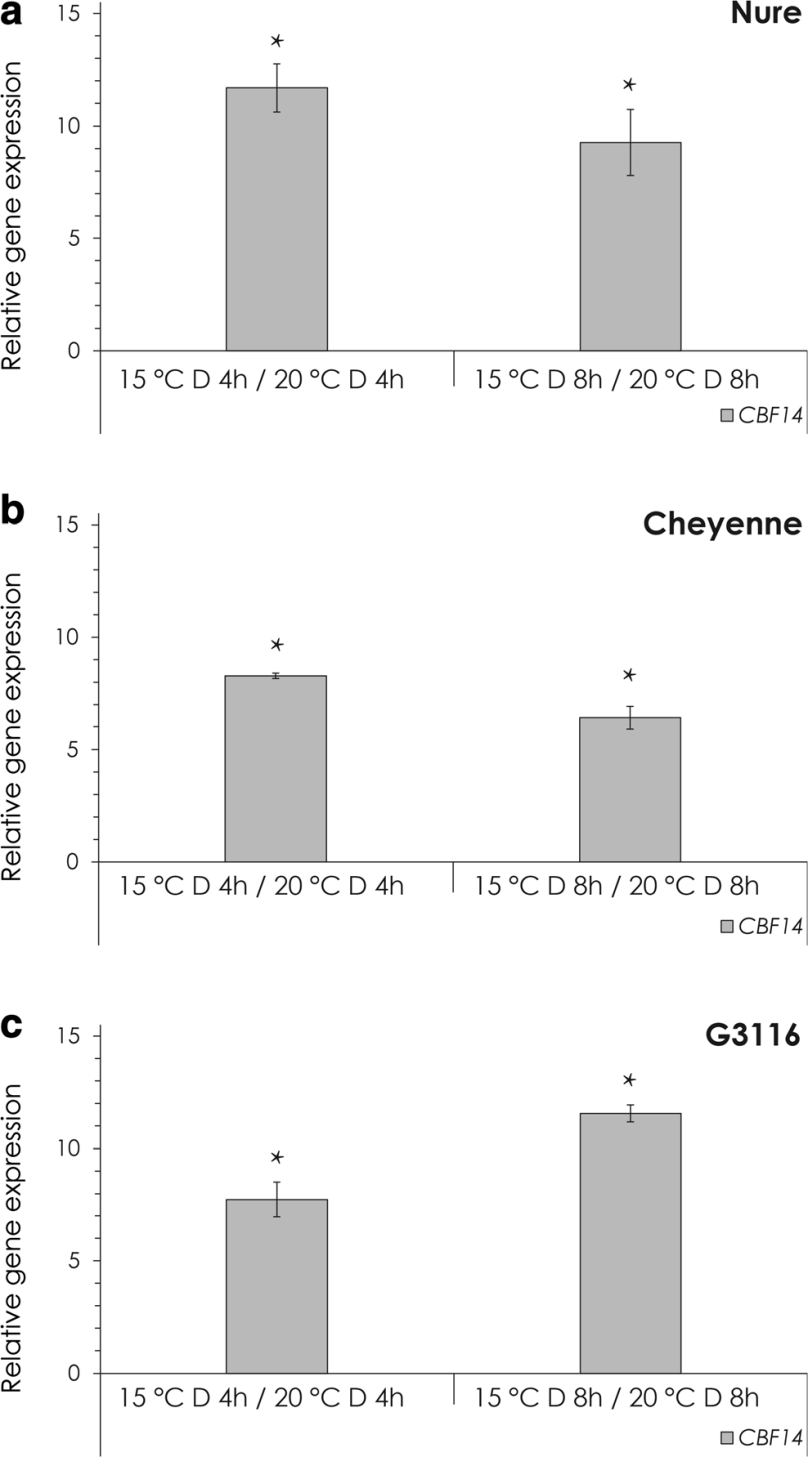
The effect of temperature on *CBF14* expression in the dark. Relative expression levels of *CBF14* in plants transferred from 20 to 15 °C for 4 or 8 h are shown. **a** Nure, **b** Cheyenne, **c** G3116. Expression levels were calculated using the ΔΔCt method and were normalized to the values from the control plants, which were kept at 20 °C for 4 or 8 h. *Asterisk*: Significant at the level of *P* < 0.05 compared with the 4- or 8-h control samples. Results of the comprehensive set of statistical analysis are shown in S2 Table

First, the temperature response of *CBF14* expression was determined in barley, wheat and einkorn. It has been demonstrated that Arabidopsis *CBF* mRNA accumulation reaches a peak 8 h after light induction at room temperature (Lee and Thomashow 2012), but cold induction culminates in a faster response with a maximum at 4–6 h after the temperature shift in barley and Triticeae (Stockinger et al. 2007; Campoli et al. 2009). Dark-adapted plants were placed from 20 to 15 °C or kept at 20 °C in the dark for 8 h. The temperature shift itself caused 6–11-fold change (**S3** Table A-C) in the level of gene expression 4 and 8 h after the cold treatment in each genotype (Fig. 1a–c).

Our previous results showed that white light triggers a very strong induction of *CBF14* expression at 15 °C in *T. monococcum* cv. G3116 (Novák et al. 2016). Similarly, the 4 or 8 h white light treatment caused a dramatic *CBF14* induction at 15 °C both in Nure and Cheyenne (**S1** Fig. A, B).

**Fig. 2:**
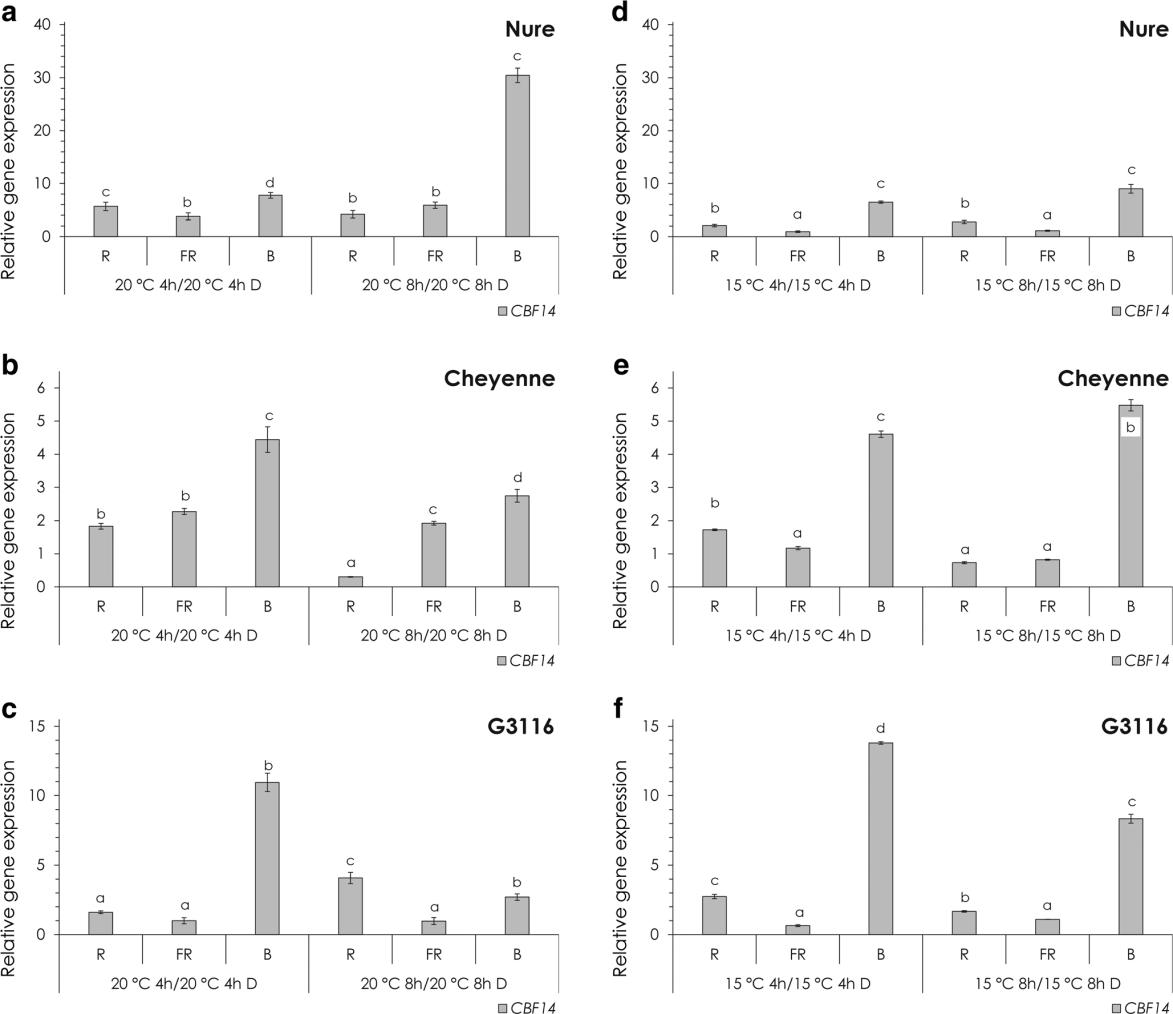
The effect of light on *CBF14* expression. **a**–**c** Relative expression of *CBF14* at 20 °C after 4 or 8 h of R, FR and B light treatment in Nure (**a**), Cheyenne (**b**) and G3116 (**c**). Control plants were kept in the dark for 4 or 8 h at 20 °C. **d**–**e** Relative expression of *CBF14* at 15 °C after 4 or 8 h of R, FR and B light treatment in Nure (**d**), Cheyenne (**e**) and G3116 (**f**). Control plants were kept in the dark for 4 or 8 h at 15 °C. *Different letters* indicate statistically different (*P* < 0.05) expression levels, where *a* represents the 4- or 8-h control treatment. Results of the comprehensive set of statistical analysis are shown in S2 Table

To characterize the wavelength dependence of this phenomenon, the effect of monochromatic light treatments on the *CBF14* expression was determined both at 20 and 15 °C in dark-adapted plants transferred to light for 4 or 8 h (Fig. 2).

In order to exclude the effect of temperature on *CBF14* expression, mRNA levels from the R, FR and B treated plants were normalized to those from the dark grown plants kept at the same temperature and harvested at the same time. Surprisingly, B light caused a considerable induction of the *CBF14* expression in every genotype similarly at both temperatures and both time points. R and FR light also induced slightly *CBF14* expression, but the magnitude of this positive influence was lower (2–5-fold) (**S4** Table A-F). Although *CBF14* expression was generally higher at 15 °C in the dark (Fig. 1), the applied monochromatic light treatments elicited similar fold changes at 20 °C and at the colder temperature in the wheat genotypes (Fig. 2b, c, e, f). In contrast, Nure *CBF14* transcription showed stronger light sensitivity at 20 °C than at 15 °C in every light conditions, especially after 8 h of B light (30-fold compared with 9-fold) illumination (Fig. 2a, d). The kinetic of the B light-induced *CBF14* expression also shows differences between species with higher transcript levels after 4 or 8 h of irradiation in wheat or barley, respectively (Fig. 2a–f).

**Fig. 3:**
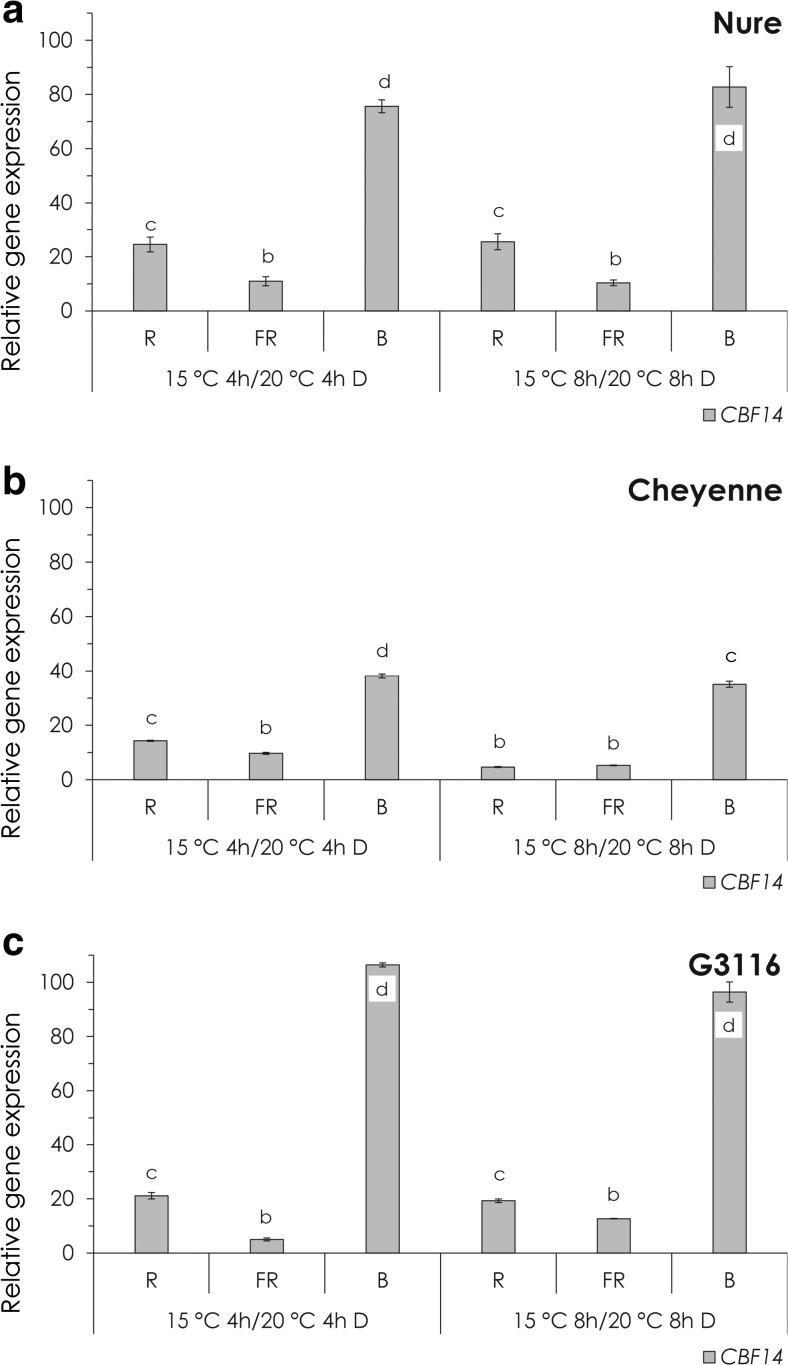
The combined effect of light and temperature on *CBF14* expression. Relative expression of *CBF14* at 15 °C after 4 or 8 h of R, FR and B light treatment in Nure (**a**), Cheyenne (**b**) and G3116 (**c**). Control plants were kept in the dark for 4 or 8 h at 20 °C. *Different letters* indicate statistically different (*P* < 0.05) expression levels, where *a* represents the 4- or 8-h control treatment. Results of the comprehensive set of statistical analysis are shown in **S2** Table

After analysing the impact of temperature shifts (Fig. 1) and monochromatic light treatments (Fig. 2) on *CBF14* expression separately, the combined effect of the temperature and light treatments was determined by the recalculation of the collected data. To this end, *CBF14* transcript levels in monochromatic light-treated plants transferred to 15 °C were normalized to *CBF14* levels in dark-adapted plants grown and kept at 20 °C (Fig. 3, **S5** Table A-C).

B light had the most pronounced effect in each genotype. The most frost tolerant Cheyenne showed the lowest (35-fold) changes in expression (Fig. 3b) compared to the less frost tolerant G3116 and Nure (80–100-fold) (Fig. 3a, c). In contrast, no extraordinary differences were found among the different genotypes when *CBF14* expression was induced by R and FR treatments. Interestingly, the effect of temperature and light induction was perfectly additive, indicating separated signalling routes of temperature and light to the level of *CBF14* expression.

Several components of the low temperature-induced CBF pathway have already been revealed, primarily in Arabidopsis (Chinnusamy et al. 2003; Agarwal et al. 2006; Badawi et al. 2008; Boldizsár et al. 2016). To collect more details about the regulation of *CBF14* gene expression by low temperature and light, we tested transcriptional responses of genes, which act in the low temperature pathway upstream of *CBF14* in Arabidopsis and possess homologs in wheat and/or barley. *ICE2* from Nure, *R2R3-MYB* and *ICE41* from Cheyenne and G3116 have been included in the experiments. Low temperature caused a 2-fold increase in *R2R3-MYB* transcript levels in Cheyenne, but had only marginal effects in all other cases (**S2** Fig). Monochromatic R, FR and B light treatment resulted in a 2–3-fold increase in *R2R3-MYB* transcript level in Cheyenne at both temperature, and FR light caused a 3-fold increase in *R2R3-MYB* transcript level in G3116 at 15 °C (**S3** Fig). However, the combined effect of B light and low temperature remained far below of those changes observed for *CBF14* gene expression in all genotypes (**S4** Fig).

### Expression of the Photoreceptor Genes

Higher plants evolved photoreceptors sensing different regions of the spectrum. Since R, FR and B light induced the expression of *CBF14*, phytochrome and cryptochrome receptors are very likely involved in this regulation. The effectiveness of the photoreceptors depends largely on the amount of the active receptors. To measure the level of the activated receptors is beyond the possibilities of our laboratory. On the other hand, the amount of the total available photoreceptor apoproteins partially depends on the corresponding transcripts levels, but there are limited data available on the light responses of these photoreceptor genes in wheat and barley genotypes. This prompted us to measure transcript levels of photoreceptor genes in response to the different temperature and light treatments that we used in the experiments above.

### Cryptochrome Expression

**Fig. 4:**
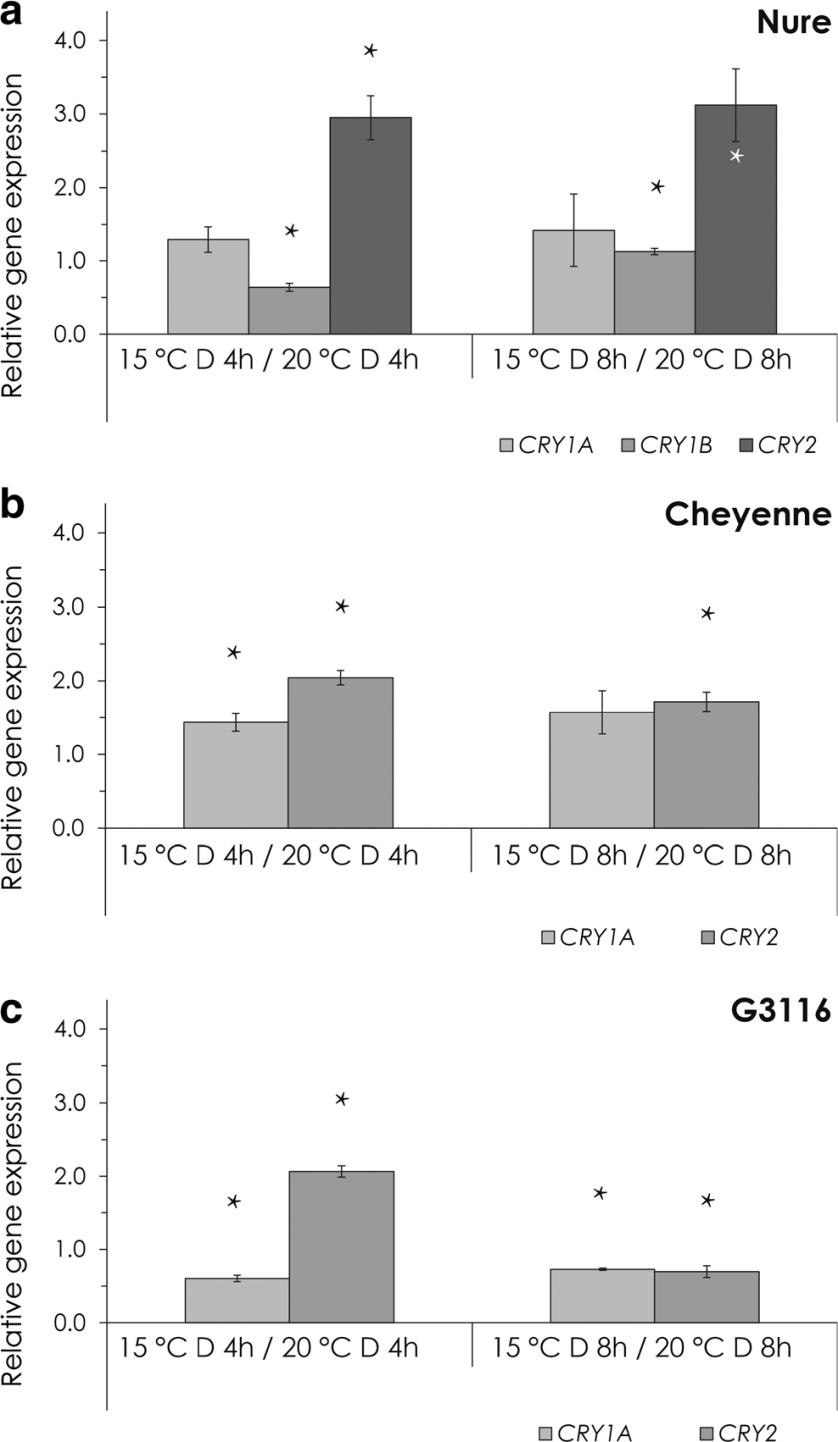
The effect of temperature on cryptochrome gene expression in the dark. Relative expression levels of *CRYs* in plants transferred from 20 to 15 °C for 4 or 8 h are shown. **a** Nure, **b** Cheyenne, **c** G3116. Expression levels were calculated using the ΔΔCt method and were normalized to the values from the control plants, which were kept at 20 °C for 4 or 8 h. *Asterisk*: Significant at the level of *P* < 0.05 compared with the 4- or 8-h control samples. Results of the comprehensive set of statistical analysis are shown in **S2** Table

Temperature drop had no effect on the *CRY* expression levels in the dark except for *CRY2*, which showed a slight (max 2–3-fold) increase in all genotypes (Fig. 4).

**Fig. 5:**
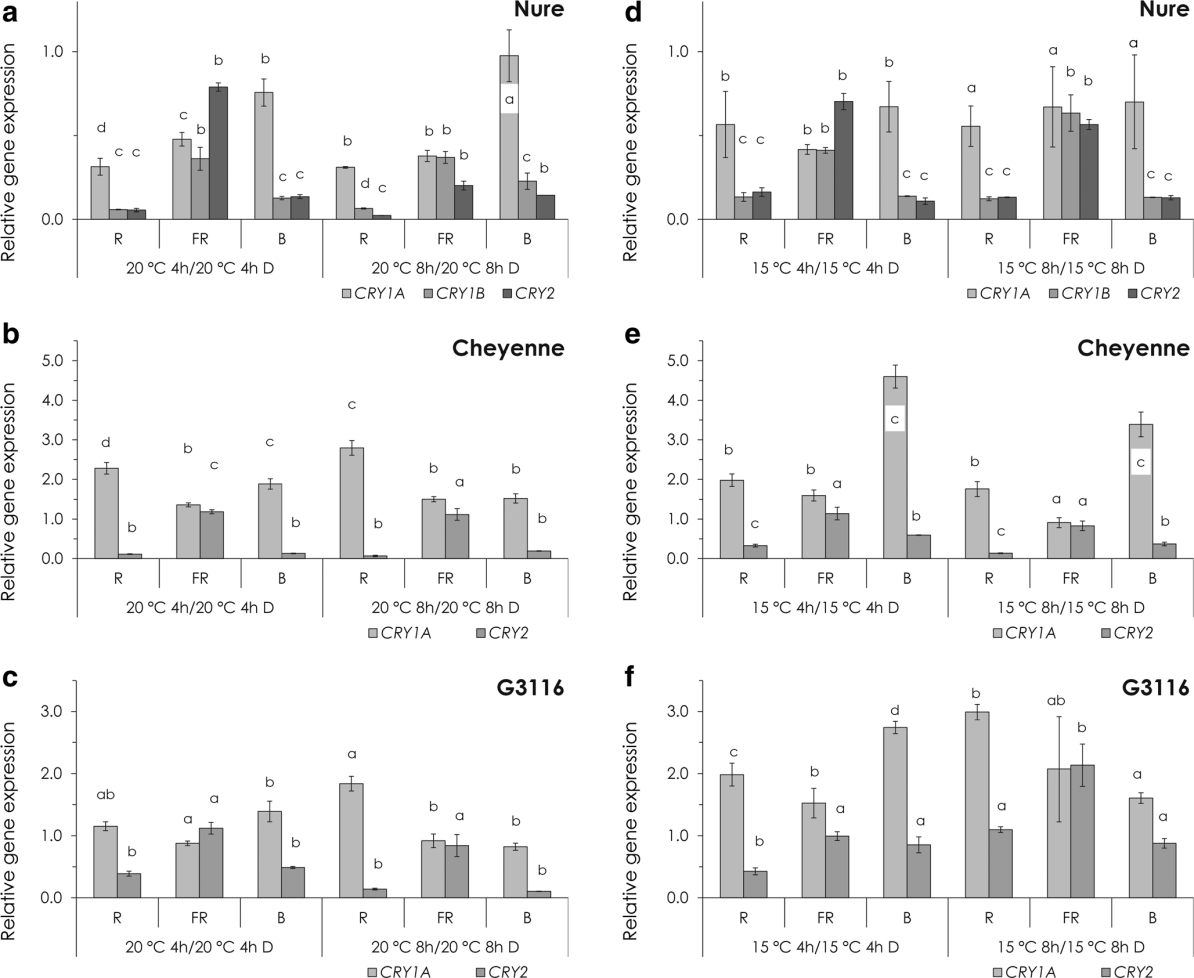
The effect of light on cryptochrome gene expression. **a**–**c** Relative expression of *CRYs* at 20 °C after 4 or 8 h of R, FR and B light treatment in Nure (**a**), Cheyenne (**b**) and G3116 (**c**). Control plants were kept in the dark for 4 or 8 h at 20 °C. **d**–**e** Relative expression of *CRYs* at 15 °C after 4 or 8 h of R, FR and B light treatment in Nure (**d**), Cheyenne (**e**) and G3116 (**f**). Control plants were kept in the dark for 4 or 8 h at 15 °C. *Different letters* indicate statistically different (*P* < 0.05) expression levels, where *a* represents the 4- or 8-h control treatment. Results of the comprehensive set of statistical analysis are shown in **S2** Table

**Fig. 6:**
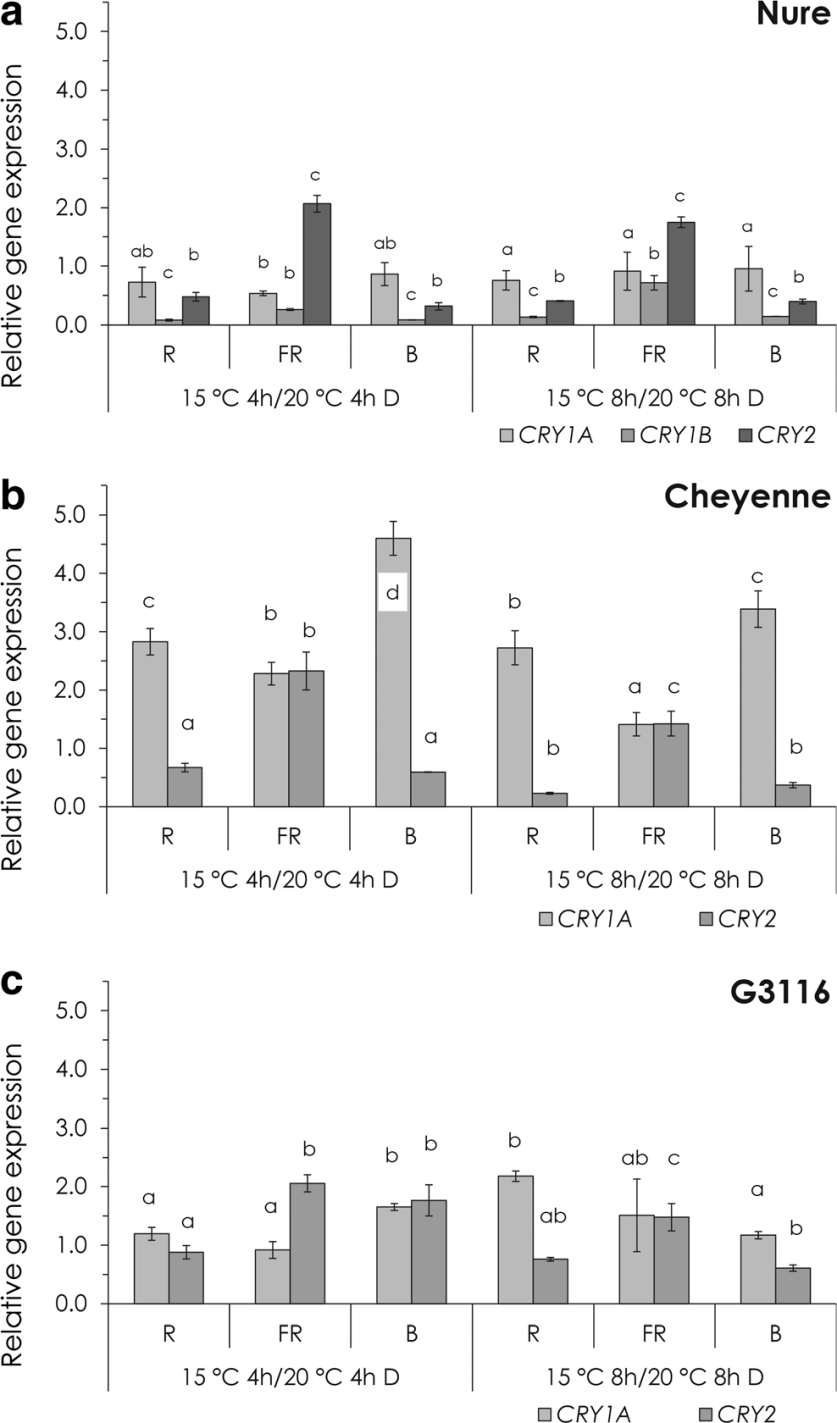
The combined effect of light and temperature on cryptochrome expression. Relative expression of *CRYs* at 15 °C after 4 or 8 h of R, FR and B light treatment in Nure (**a**), Cheyenne (**b**) and G3116 (**c**). Control plants were kept in the dark for 4 or 8 h at 20 °C. *Different letters* indicate statistically different (*P* < 0.05) expression levels, where *a* represents the 4- or 8-h control treatment. Results of the comprehensive set of statistical analysis are shown in **S2** Table

Light generally had an inhibitory effect on the expression of *CRY* genes, with the exception of the wheat genotypes, where *CRY1a* showed a slight increase, especially in R light at 20 °C (Fig. 5b, c) and in B light at 15 °C (Fig. 5e, f). In FR light, *CRY1a* showed the same expression level like in the dark (Fig. 5b, c, e, f). The wheat *CRY2* mRNA levels were reduced by R and B light in a temperature-independent way, and similarly to *CRY1a*, FR light did not affect the expression (Fig. 5b, c, e, f). Expression of the Nure *CRY1b* and *CRY2* genes showed the same tendency: reduced levels by R and B and a slighter decrease by FR light treatment, independent of the temperature (Fig. 5a, d). The barley *CRY1a* was the less sensitive to monochromatic light (Fig. 5a, d). Similarly to the regulation of *CBF14*, the effects of temperature and light treatments on *CRY* gene expression were additive (Fig. 6).

### Phytochrome Expression

Temperature shift had only a very small effect on the expression level of phyA, B and C in the dark (**S5** Fig). In contrast, monochromatic light treatments repressed phytochrome gene expression in most cases (**S6** Fig). Particularly, massive inhibition was observed in Nure after R and B light treatments at both temperatures. FR light caused a smaller decrease in a temperature-independent manner (**S6** Fig. A, D). Phytochrome expression in the wheat genotypes decreased to a greater or lesser extent in response to all light treatments, among which FR light at 20 °C was the most effective in Cheyenne (**S6** Fig. B). Since temperature had no effect on phytochrome gene expression, the combined effect of temperature shifts and light treatments reflected the previously detected light repression, which was outstanding in Nure, but less remarkable in Cheyenne and G3116 (**S7** Fig).

## Discussion

*CBF14* can be induced by white light, activated by low temperature and it is responsible for the initiation of freezing tolerance (Stockinger et al. 2007; Campoli et al. 2009; Vashegyi et al. 2013; Dhillon and Stockinger 2013). White light triggers a very strong *CBF14* induction at 15 °C in G3116 (Novák et al. 2016) and also in Nure and Cheyenne (**S1** Fig). It has also been observed that light quality, especially low R/FR ratio, can induce *CBF14* expression and freezing tolerance in a temperature- and genotype-dependent manner through the phytochrome system, where phyB has a negative while phyA has a potential positive effect on *CBF14* transcription (Novák et al. 2016; Wang et al. 2016). The effect of light quality on *CBF14* expression in the same winter wheat and barley cultivars was further analysed in the present study.

The most direct way to determine the contribution of the different wavelength-specific photoreceptors to the light regulation of *CBF14* expression would be to include photoreceptor mutants in the experiments. Unfortunately, such mutants were not available in the genotypes we analysed, prompting us to treat the plants with monochromatic light, which is selectively and specifically absorbed by a given photoreceptor. White light was divided into three biologically active segments: the B, R and FR regions were chosen to induce the cryptochrome and the phytochrome system at two temperatures.

The effect of light and low temperature on *CBF14* expression was tested separately and in combination as well (Figs. 1, 2 and 3). Both environmental factors had a positive role on *CBF14* expression separately. We could detect a 6–11-fold induction caused by low temperature, independent of the genotype. Monochromatic light treatments resulted in a 1–32-fold induction, depending on the genotype and wavelength of the light used, but the actual magnitude of induction was largely independent of temperature.

Interestingly, *CBF14* expression showed the highest sensitivity to B light and was much less responsive to R and FR light in all the three genotypes. However, as a significant genotype-specific difference, we found that the barley genotype Nure was more sensitive to any type of monochromatic light at 20 °C, especially to the B illumination. Based on the relevant data available from the model plant Arabidopsis, the obvious candidates for mediating the B light signal are the CRY1/2 and the phyA photoreceptors. Considering the facts that (i) B light treatments caused much more pronounced effects on *CBF14* expression as compared with FR light and that (ii) phyA is also effectively activated by FR light, we concluded that the CRY photoreceptors play the prominent role in this response.

It is well known that relatively small changes in the expression level of phytochromes can influence particular light responses very strongly (Cherry et al. 1992).

We showed that low temperature had only marginal effects on the expression of phytochrome genes in cereals, which is consistent with the results from Arabidopsis (Jung et al. 2016). To reveal how light regulates the abundance of these photoreceptors, one could analyse the light-induced changes at transcriptional, translational or post-translational levels.

In Arabidopsis, light regulates the protein stability of the five phytochromes, although to a different extent (Nagy and Schafer 2002; Casal et al. 2013). In cereals, the TaCRY2 protein is located in the nucleus in dark and it is degraded by B light (Xu et al. 2009). The effectiveness of photoreceptor-initiated signalling largely depends on the amount of the active receptors; thus, ectopic overexpression usually confers hypersensitivity to light. Since the measurement of the total amount or the proportion of the activated receptors requires special laboratory instrumentation, we monitored transcription of photoreceptor genes in response to different monochromatic light treatments, which has not been tested in details in cereals yet.

In Arabidopsis, the *PHYA* transcript shows decreased abundance in light-grown seedlings (Casal et al. 2013). Even stronger light-induced down-regulation of *phyA* has been observed in monocots (Kay et al. 1989; Baba-Kasai et al. 2014). Arabidopsis *CRY1/2* genes are expressed ubiquitously in all cell types and organs examined, and *CRY* mRNA levels are not dramatically affected by B light (Yu et al. 2010). In contrast, the pea *CRY2b* gene is repressed by B light illumination (Platten et al. 2005), which is consistent with our results (Fig. 5). Expression level of *TaCRY1a* is induced by R light, and the TaCRY1a-GFP fusion protein is transferred from the nucleus to the cytoplasm in response to B light (Xu et al. 2009). B light illumination induced the protein abundance of CRY1 in *Brassica napus* (Chatterjee et al. 2006). In our study, the wheat (*Ta*) *CRY1a* gene is slightly R and B light inducible (Fig. 5b, c, e, f), but the barley *CRY1a* is repressed by any kind of light treatments (Fig. 5a, d). However, it is very unlikely that the mild B light-induced change in *CRY1/CRY2* expression contributes significantly to the massive transcriptional induction of *CBF14*, indicating the role of the signalling pathway, which connects the activated CRY receptors with the promoter of *CBF14*.

We can also conclude that the homolog genes, acting in the low temperature-induced pathway in Arabidopsis upstream of *CBF14*, play no or only marginal role in mediating cold and/or light signals to the *CBF14* promoters in wheat and barley.

Our results demonstrate that the effects of monochromatic light treatments and low temperature on *CBF14* gene expression are almost quantitatively additive. This observation indicates that the integration of the two signalling routes, relaying the effect of light and temperature to the level of *CBF14* transcription, may occur at one of the terminal steps of signal transduction, probably at the activation of the promoter of *CBF14*. In order to shed light on the molecular mechanism by which the integration of the two most significant environmental signals takes place, future work should focus on the identification and functional analysis of *cis*-elements and the corresponding transcription factors controlling the activation of the *CBF14* promoter in response to light and low temperature in wheat and barley.

## Electronic supplementary material

S1 Table **Sequence of oligonucleotides used in this study. (DOCX 22 kb)**

S2 Table **Comprehensive statistical evaluation of expression data presented in this study. All the temperature and light conditions used in this study were compared by the Tukey’s b method. Different letters represent significantly different groups** (*P* < 0.05) **within samples belonging to the same timepoint, where least mean value is represented by ‘a’. (DOCX 16 kb)**

S3 Table **Gene expression data of temperature treated Nure (A), Cheyenne (B) and G3116 (C) plants.** Relative gene expression levels (+/− SD) in plants transferred from 20 °C to 15 °C for 4 or 8 h. Expression levels were calculated using the ΔΔCt method and normalised to the values from the control plants, which were kept at 20 °C for 4 or 8 h. All plants were kept in darkness. (DOCX 15 kb)

S4 Table **Gene expression data of light treated Nure (A, B), Cheyenne (C, D) and G3116 (E, F) plants.** Relative expression at 20 °C after 4 or 8 h of R, FR and B light treatment in Nure (A), Cheyenne (C) and G3116 (E). Control plants were kept in the dark for 4 or 8 h at 20 °C. Relative expression (+/− SD) at 15 °C after 4 or 8 h of R, FR and B light treatment in Nure (B), Cheyenne (D) and G3116 (F). Control plants were kept in the dark for 4 or 8 h at 15 °C. (DOCX 24 kb)

S5 Table **Combined effect of light and temperature on gene expression levels in Nure (A), Cheyenne (B) and G3116 (C) plants.** Relative expression levels (+/− SD) at 15 °C after 4 or 8 h of R, FR and B light treatment are shown, where control plants were kept in the dark for 4 or 8 h at 20 °C. (DOCX 20 kb)

S1 Fig **The effect of white light on** ***CBF14*** **expression**. Relative expression of *CBF14* at 20 °C or 15 °C after 4 or 8 h of white light treatment in Nure (A), Cheyenne (B). Expression levels presented were calculated using the ΔΔCt method, where Ct values were normalized to the Ct values of house-keeping genes (*cyclophilin* for panel A and *phosphogluconate dehydrogenase* for panel B). (GIF 15 kb)

High resolution image (TIFF 480 kb)

S2 Fig **The effect of temperature on** ***ICE2, R2R3-MYB and ICE41*** **gene expression in the dark.** Relative gene expression levels in plants transferred from 20 °C to 15 °C for 4 or 8 h are shown. A) Nure, B) Cheyenne, C) G3116. Expression levels were calculated using the ΔΔCt method and were normalised to the values from the control plants, which were kept at 20 °C for 4 or 8 h. * Significant at the level of *P* < 0.05 compared with the 4- or 8-h control samples. Results of the comprehensive set of statistical analysis are shown in S2.Table (GIF 99 kb)

High resolution image (TIFF 1917 kb)

S3 Fig **The effect of light on** ***ICE2, R2R3MYB*** **and** ***ICE41*** **gene expression.** A-C) Relative gene expressions at 20 °C after 4 or 8 h of R, FR and B light treatment in Nure (A), Cheyenne (B) and G3116 (C). Control plants were kept in the dark for 4 or 8 h at 20 °C. D-E) Relative expression at 15 °C after 4 or 8 h of R, FR and B light treatment in Nure (D), Cheyenne (E) and G3116 (F). Control plants were kept in the dark for 4 or 8 h at 15 °C. Different letters indicate statistically different (*P* < 0.05) expression levels, where ‘a’ represents the 4- or 8-h control treatment. Results of the comprehensive set of statistical analysis are shown in S2.Table (GIF 191 kb)

High resolution image (TIFF 2497 kb)

S4 Fig **The combined effect of light and temperature on** ***ICE2, R2R3MYB*** **and** ***ICE41*** **gene expression.** Relative expression levels at 15 °C after 4 or 8 h of R, FR and B light treatment in Nure (A), Cheyenne (B) and G3116 (C). Control plants were kept in the dark for 4 or 8 h at 20 °C. Different letters indicate statistically different (*P* < 0.05) expression levels, where ‘a’ represents the 4- or 8-h control treatment. Results of the comprehensive set of statistical analysis are shown in S2.Table (GIF 93 kb)

High resolution image (TIFF 1545 kb)

S5 Fig **The effect of temperature on phytochrome gene expression in the dark.** Relative expression levels of *PHYs* in plants transferred from 20 °C to 15 °C for 4 or 8 h are shown. A) Nure, B) Cheyenne, C) G3116. Expression levels were calculated using the ΔΔCt method and were normalised to the values from the control plants, which were kept at 20 °C for 4 or 8 h. * Significant at the level of *P* < 0.05 compared with the 4- or 8-h control samples. (GIF 29 kb)

High resolution image (TIFF 1208 kb)

S6 Fig **The effect of light on phytochrome gene expression.** A-C) Relative expression of *PHYs* at 20 °C after 4 or 8 h of R, FR and B light treatment in Nure (A), Cheyenne (B) and G3116 (C). Control plants were kept in the dark for 4 or 8 h at 20 °C. D-E) Relative expression of *PHYs* at 15 °C after 4 or 8 h of R, FR and B light treatment in Nure (D), Cheyenne (E) and G3116 (F). Control plants were kept in the dark for 4 or 8 h at 15 °C. Different letters indicate statistically different (*P* < 0.05) expression levels, where ‘a’ represents the 4- or 8-h control treatment. (GIF 67 kb)

High resolution image (TIFF 1817 kb)

S7 Fig **The combined effect of light and temperature on phytochrome gene expression.** Relative expression of *PHYs* at 15 °C after 4 or 8 h of R, FR and B light treatment in Nure (A), Cheyenne (B) and G3116 (C). Control plants were kept in the dark for 4 or 8 h at 20 °C. Different letters indicate statistically different (*P* < 0.05) expression levels, where ‘a’ represents the 4- or 8-h control treatment. (GIF 35 kb)

High resolution image (TIFF 1236 kb)
