# Supplementary material for: Light and Temperature Signalling at the Level of CBF14 Gene Expression in Wheat and Barley
Source: Plant Mol Biol Report. 2017 May 12;35(4):399–408. doi: 10.1007/s11105-017-1035-1 (PMC5504222; doi:10.1007/s11105-017-1035-1)
Supplement: Supplementary file 1 — Sequence of oligonucleotides used in this study. (DOCX 22 kb) [file 11105_2017_1035_MOESM1_ESM.docx]

| Primer sequences and references | | | |
| --- | --- | --- | --- |
| **Name of the gene** | **Forward sequences 5’-3’** | **Reverse sequences 5’-3’** | **references** |
| CBF14 | CCACCAAATATGGGAGGAAA | CTTTCACAATGAACGAGCA | Dhillon et al. 2010 |
| CRY1a | CAGCCTCAGACTCGGATGG | CTGAAGTGGATGGTGCTATGCC | EF601539.1  EF601540.1 |
| CRY2 | CTGGTGGTTATTTAGTTGGGTCGT | GTACCACTATCGGCACTGTCATC | EF601541.1  EF601542.1 |
| Cyclophilin | CCTGTCGTGTCGTCGGTCTAAA | ACGCAGATCCAGCAGCCTAAAG | Burton et al. 2004 |
| HvCBF14 | GTTGAATGAGCACTGGTTTGG | ACAATGAACGAGCAGGTATGG | Morran et al. 2011 |
| HvCRY1a | CACATGGAAGTGGACCGTGC | CGCTGGCAACTTGTTCCCTG | DQ201149.1  DQ201150.1  DQ201151.1 |
| HvCRY1b | GATGGAAGTCCATTGGGAACC | CATACGGTGCTGAGGTTGCTG | DQ201152.1  DQ201153.1  DQ201154.1 |
| HvCRY2 | GGCTCGCGAGTGCTTAGATG | GGAGCACTTGGAGATAGTTCCTTC | DQ201155.1  DQ201156.1 |
| PHYA | TGCAGCACATTCAGAGAGGG | CCCTAGTGCCTTGTGCAGAG | Novák et al. 2016 |
| PHYB | GACCTGTCGCCTCACCACT | CAGCGAGATCTCCCGTGC | Novák et al. 2016 |
| PHYC | TCCAAGCACAGCGAGCG | GGTTGACCTCCCCGAAGAC | Novák et al. 2016 |
| R2R3-MYB | TTGGTCGTCGATCCTCCACAG | GAGGAGAAGAAGGCGGAGGTG | Boldizsár et al. 2016 |
| ICE41 | GCAACCCCTTCAACGTCGA | ACCACGGCATTCTCCAGC | EU562183.1 |
| HvICE2 | ACGGGAAGGGCAAGAAGAAG | TTGCGTCACCGAGGATTG | DQ113909.1 |
| Ta30797 | GCCGTGTCCATGCCAGTG | TTAGCCTGAACCACCTGTGC | Paolacci et al. 2009 |
