# Supplementary material for: Light and Temperature Signalling at the Level of CBF14 Gene Expression in Wheat and Barley
Source: Plant Mol Biol Report. 2017 May 12;35(4):399–408. doi: 10.1007/s11105-017-1035-1 (PMC5504222; doi:10.1007/s11105-017-1035-1)
Supplement: Supplementary file 2 — Comprehensive statistical evaluation of expression data presented in this study. All the temperature and light conditions used in this study were compared by the Tukey’s b method. Different letters represent significantly different groups (P < 0.05) within samples belonging to the same timepoint, where least mean value is represented by ‘a’. (DOCX 16 kb) [file 11105_2017_1035_MOESM2_ESM.docx]

| CHEYENNE | CBF14 | | CRY1a | | CRY2 | | PHYA | | PHYB | | PHYC | | MYB20 | | ICE41 | |
| --- | --- | --- | --- | --- | --- | --- | --- | --- | --- | --- | --- | --- | --- | --- | --- | --- |
|  | 4h | 8h | 4h | 8h | 4h | 8h | 4h | 8h | 4h | 8h | 4h | 8h | 4h | 8h | 4h | 8h |
| 20 °C R | a | a | b | b | a | a | bc | a | bc | abc | d | c | b | d | a | a |
| 20 °C FR | a | b | a | a | c | c | ab | c | a | a | a | bc | c | c | b | bc |
| 20 °C B | b | b | b | a | a | ab | abc | a | cd | a | b | a | a | b | b | a |
| 15 °C D | c | d | a | a | d | e | abc | d | bc | c | cd | d | d | b | b | bc |
| 15 °C R | e | c | c | b | b | ab | abc | ab | a | bc | a | bc | bc | a | ab | ab |
| 15 °C FR | d | c | b | a | d | d | abc | bc | ab | ab | a | a | e | d | c | c |
| 15 °C B | f | e | d | c | b | b | c | abc | d | d | bc | ab | a | d | b | c |
|  |  |  |  |  |  |  |  |  |  |  |  |  |  |  |  |  |
| G3116 | CBF14 | | CRY1a | | CRY2 | | PHYA | | PHYB | | PHYC | | MYB20 | | ICE41 | |
|  | 4h | 8h | 4h | 8h | 4h | 8h | 4h | 8h | 4h | 8h | 4h | 8h | 4h | 8h | 4h | 8h |
| 20 °C R | a | a | c | cd | a | a | b | bc | a | a | ab | e | e | d | a | a |
| 20 °C FR | a | a | b | ab | b | b | c | a | cd | a | b | bc | ab | cd | c | a |
| 20 °C B | d | a | c | a | a | a | a | a | cd | a | a | ab | c | ab | abc | a |
| 15 °C D | c | b | a | a | c | b | b | d | d | b | ab | d | c | a | abc | a |
| 15 °C R | e | c | c | d | b | b | b | c | bc | c | a | e | a | b | d | b |
| 15 °C FR | b | b | b | bc | c | c | b | a | ab | a | a | a | d | e | ab | a |
| 15 °C B | f | d | d | ab | c | b | b | ab | d | b | ab | c | bc | c | bc | a |
|  |  |  |  |  |  |  |  |  |  |  |  |  |  |  |  |  |
| NURE | CBF14 | | CRY1a | | CRY1b | | CRY2 | | PHYA | | PHYB | | PHYC | | ICE2 | |
|  | 4h | 8h | 4h | 8h | 4h | 8h | 4h | 8h | 4h | 8h | 4h | 8h | 4h | 8h | 4h | 8h |
| 20 °C R | a | a | a | a | a | a | a | a | a | a | a | a | a | a | ab | a |
| 20 °C FR | a | a | ab | a | c | c | c | a | c | ab | b | bc | a | a | c | b |
| 20 °C B | ab | b | bc | ab | a | b | a | a | a | a | a | a | a | a | ab | a |
| 15 °C D | b | a | d | b | d | e | e | c | d | b | d | c | b | b | d | c |
| 15 °C R | c | b | bc | ab | a | ab | b | a | a | a | a | a | a | a | ab | a |
| 15 °C FR | b | a | abc | ab | b | d | d | b | b | ab | c | ab | a | a | b | b |
| 15 °C B | d | c | c | ab | a | ab | ab | a | a | a | a | a | a | a | a | a |
