# Supplementary material for: Light and Temperature Signalling at the Level of CBF14 Gene Expression in Wheat and Barley
Source: Plant Mol Biol Report. 2017 May 12;35(4):399–408. doi: 10.1007/s11105-017-1035-1 (PMC5504222; doi:10.1007/s11105-017-1035-1)
Supplement: Supplementary file 3 — Gene expression data of temperature treated Nure (A), Cheyenne (B) and G3116 (C) plants. Relative gene expression levels (+/− SD) in plants transferred from 20 °C to 15 °C for 4 or 8 h. Expression levels were calculated using the ΔΔCt method and normalised to the values from the control plants, which were kept at 20 °C for 4 or 8 h. All plants were kept in darkness. (DOCX 15 kb) [file 11105_2017_1035_MOESM3_ESM.docx]

| Gene expression data of dark treated Nure plants | | | | | | | | | |
| --- | --- | --- | --- | --- | --- | --- | --- | --- | --- |
| **A** | CBF14 | PHYA | | PHYB | PHYC | CRY1A | | CRY1B | CRY2 |
| 15°C D 4h/20°C D 4h | 11,69±1,06 | 0,43±0,02 | | 1,42±0,06 | 1,66±0,26 | 1,29±0,18 | | 0,64±0,05 | 2,95±0,30 |
| 15°C D 8h/20°C D 8h | 9,27±1,47 | 0,85±0,04 | | 1,24±0,10 | 0,89±0,11 | 1,42±0,50 | | 1,13±0,04 | 3,12±0,49 |
| Gene expression data of dark treated Cheyenne plants | | | | | | | | |  |
| **B** | CBF14 | PHYA | PHYB | | PHYC | | CRY1A | CRY2 |  |
| 15 °C D 4h/20 °C D 4h | 8,28±0,12 | 1,03±0,14 | 0,86±0,13 | | 1,15±0,15 | | 1,43±0,12 | 2,04±0,10 |  |
| 15 °C D 8h/20 °C D 8h | 6,41±0,51 | 0,65±0,01 | 1,25±0,20 | | 1,97±0,10 | | 1,57±0,29 | 1,72±0,13 |  |
| Gene expression data of dark treated G3116 plants | | | | | | | | |  |
| **C** | CBF14 | PHYA | PHYB | | PHYC | | CRY1A | CRY2 |  |
| 15 °C D 4h/20 °C D 4h | 7,74±0,78 | 0,78±0,04 | 0,69±0,05 | | 0,60±0,04 | | 0,60±0,04 | 2,07±0,08 |  |
| 15 °C D 8h/20 °C D 8h | 11,56±0,37 | 1,29±0,24 | 0,97±0,15 | | 0,77±0,07 | | 0,73±0,02 | 0,70±0,08 |  |
