# Supplementary material for: Light and Temperature Signalling at the Level of CBF14 Gene Expression in Wheat and Barley
Source: Plant Mol Biol Report. 2017 May 12;35(4):399–408. doi: 10.1007/s11105-017-1035-1 (PMC5504222; doi:10.1007/s11105-017-1035-1)
Supplement: Supplementary file 4 — Gene expression data of light treated Nure (A, B), Cheyenne (C, D) and G3116 (E, F) plants. Relative expression at 20 °C after 4 or 8 h of R, FR and B light treatment in Nure (A), Cheyenne (C) and G3116 (E). Control plants were kept in the dark for 4 or 8 h at 20 °C. Relative expression (+/− SD) at 15 °C after 4 or 8 h of R, FR and B light treatment in Nure (B), Cheyenne (D) and G3116 (F). Control plants were kept in the dark for 4 or 8 h at 15 °C. (DOCX 24 kb) [file 11105_2017_1035_MOESM4_ESM.docx]

| Gene expression data of light treated Nure plants 20°C | | | | | | | | |
| --- | --- | --- | --- | --- | --- | --- | --- | --- |
| **A** |  | CBF14 | PHYA | PHYB | PHYC | CRY1A | CRY1B | CRY2 |
| 20 °C 4h/ 20 °C 4h D | R | 5,67±0,79 | 0,00±0,00 | 0,02±0,00 | 0,01±0,00 | 0,31±0,05 | 0,06±0,00 | 0,05±0,01 |
|  | FR | 3,79±0,66 | 0,17±0,01 | 0,29±0,02 | 0,17±0,01 | 0,48±0,04 | 0,36±0,07 | 0,79±0,02 |
|  | B | 7,76±0,54 | 0,00±0,00 | 0,06±0,00 | 0,02±0,00 | 0,76±0,08 | 0,13±0,01 | 0,13±0,01 |
| 20 °C 8h/ 20 °C 8h D | R | 4,18±0,71 | 0,00±0,00 | 0,04±0,01 | 0,01±0,00 | 0,31±0,01 | 0,06±0,00 | 0,02±0,00 |
|  | FR | 5,89±0,59 | 0,28±0,03 | 0,63±0,27 | 0,12±0,01 | 0,38±0,03 | 0,37±0,04 | 0,20±0,03 |
|  | B | 30,43±1,34 | 0,00±0,00 | 0,05±0,00 | 0,01±0,00 | 0,98±0,15 | 0,23±0,05 | 0,14±0,00 |
| Gene expression data of light treated Nure plants 15°C | | | | | | | | |
| **B** |  | CBF14 | PHYA | PHYB | PHYC | CRY1A | CRY1B | CRY2 |
| 15 °C 4h/ 15 °C 4h D | R | 2,10±0,23 | 0,01±0,00 | 0,01±0,00 | 0,01±0,00 | 0,57±0,20 | 0,13±0,03 | 0,16±0,03 |
|  | FR | 0,94±0,14 | 0,19±0,02 | 0,25±0,01 | 0,08±0,01 | 0,42±0,03 | 0,41±0,02 | 0,70±0,05 |
|  | B | 6,49±0,20 | 0,02±0,00 | 0,02±0,00 | 0,01±0,00 | 0,67±0,15 | 0,14±0,00 | 0,11±0,02 |
| 15 °C 8h/ 15 °C 8h D | R | 2,78±0,32 | 0,00±0,00 | 0,02±0,00 | 0,01±0,00 | 0,56±0,12 | 0,12±0,01 | 0,13±0,00 |
|  | FR | 1,13±0,11 | 0,21±0,02 | 0,29±0,01 | 0,13±0,01 | 0,67±0,24 | 0,63±0,11 | 0,57±0,03 |
|  | B | 9,00±0,82 | 0,00±0,00 | 0,05±0,01 | 0,01±0,00 | 0,70±0,28 | 0,13±0,00 | 0,13±0,01 |
| Gene expression data of light treated Cheyenne plants 20°C | | | | | | | | |
| **C** |  | CBF14 | PHYA | PHYB | PHYC | CRY1A | CRY2 |  |
| 20 °C 4h/ 20 °C 4h D | R | 1,83±0,09 | 1,18±0,58 | 0,95±0,17 | 1,30±0,25 | 2,28±0,15 | 0,11±0,01 |  |
|  | FR | 2,27±0,09 | 0,48±0,14 | 0,39±0,07 | 0,45±0,05 | 1,36±0,05 | 1,18±0,05 |  |
|  | B | 4,44±0,39 | 0,63±0,05 | 1,04±0,15 | 0,75±0,02 | 1,88±0,13 | 0,13±0,01 |  |
| 20 °C 8h/ 20 °C 8h D | R | 0,30±0,01 | 0,18±0,03 | 0,72±0,11 | 0,53±0,11 | 2,79±0,19 | 0,07±0,02 |  |
|  | FR | 1,92±0,06 | 0,40±0,12 | 0,42±0,03 | 0,45±0,07 | 1,50±0,07 | 1,12±0,15 |  |
|  | B | 2,74±0,19 | 0,17±0,06 | 0,45±0,02 | 0,22±0,01 | 1,52±0,12 | 0,19±0,01 |  |
| Gene expression data of light treated Cheyenne plants 15°C | | | | | | | | |
| **D** |  | CBF14 | PHYA | PHYB | PHYC | CRY1A | CRY2 |  |
| 15 °C 4h/ 15 °C 4h D | R | 1,73±0,02 | 0,31±0,05 | 0,49±0,25 | 0,28±0,01 | 1,98±0,16 | 0,33±0,04 |  |
|  | FR | 1,17±0,05 | 0,62±0,11 | 0,64±0,18 | 0,30±0,04 | 1,60±0,14 | 1,14±0,16 |  |
|  | B | 4,61±0,10 | 1,32±0,41 | 1,39±0,22 | 0,92±0,08 | 4,60±0,29 | 0,59±0,01 |  |
| 15 °C 8h/ 15 °C 8h D | R | 0,73±0,03 | 0,38±0,02 | 0,82±0,03 | 0,20±0,01 | 1,76±0,19 | 0,13±0,01 |  |
|  | FR | 0,82±0,02 | 0,55±0,04 | 0,40±0,08 | 0,12±0,01 | 0,91±0,13 | 0,83±0,12 |  |
|  | B | 5,48±0,17 | 0,28±0,03 | 1,86±0,53 | 0,34±0,03 | 3,39±0,31 | 0,37±0,04 |  |
| Gene expression data of light treated G3116 plants 20°C | | | | | | | | |
| **E** |  | CBF14 | PHYA | PHYB | PHYC | CRY1A | CRY2 |  |
| 20 °C 4h/ 20 °C 4h D | R | 1,61±0,10 | 0,71±0,19 | 0,30±0,07 | 0,61±0,12 | 1,15±0,07 | 0,39±0,04 |  |
|  | FR | 0,99±0,22 | 1,26±0,07 | 0,58±0,02 | 0,72±0,04 | 0,88±0,04 | 1,12±0,09 |  |
|  | B | 10,94±0,66 | 0,34±0,02 | 0,61±0,09 | 0,54±0,04 | 1,39±0,17 | 0,49±0,02 |  |
| 20 °C 8h/ 20 °C 8h D | R | 4,08±0,40 | 0,76±0,07 | 0,65±0,09 | 1,21±0,14 | 1,84±0,12 | 0,14±0,01 |  |
|  | FR | 0,97±0,25 | 0,42±0,05 | 0,49±0,09 | 0,33±0,03 | 0,92±0,11 | 0,84±0,18 |  |
|  | B | 2,70±0,23 | 0,48±0,01 | 0,53±0,06 | 0,27±0,03 | 0,82±0,06 | 0,11±0,00 |  |
| Gene expression data of light treated G3116 plants 15°C | | | | | | | | |
| **F** |  | CBF14 | PHYA | PHYB | PHYC | CRY1A | CRY2 |  |
| 15 °C 4h/ 15 °C 4h D | R | 2,74±0,15 | 1,07±0,03 | 0,70±0,03 | 0,81±0,11 | 1,98±0,18 | 0,43±0,06 |  |
|  | FR | 0,65±0,07 | 0,80±0,01 | 0,57±0,03 | 0,79±0,02 | 1,53±0,24 | 0,99±0,07 |  |
|  | B | 13,80±0,10 | 0,85±0,07 | 0,97±0,12 | 0,97±0,08 | 2,74±0,10 | 0,85±0,13 |  |
| 15 °C 8h/ 15 °C 8h D | R | 1,67±0,06 | 0,74±0,03 | 1,37±0,10 | 1,40±0,09 | 2,99±0,13 | 1,10±0,05 |  |
|  | FR | 1,10±0,01 | 0,24±0,07 | 0,50±0,06 | 0,18±0,03 | 2,07±0,85 | 2,13±0,34 |  |
|  | B | 8,34±0,32 | 0,45±0,05 | 0,90±0,08 | 0,57±0,04 | 1,61±0,09 | 0,88±0,08 |  |
