# Supplementary material for: Light and Temperature Signalling at the Level of CBF14 Gene Expression in Wheat and Barley
Source: Plant Mol Biol Report. 2017 May 12;35(4):399–408. doi: 10.1007/s11105-017-1035-1 (PMC5504222; doi:10.1007/s11105-017-1035-1)
Supplement: Supplementary file 5 — Combined effect of light and temperature on gene expression levels in Nure (A), Cheyenne (B) and G3116 (C) plants. Relative expression levels (+/− SD) at 15 °C after 4 or 8 h of R, FR and B light treatment are shown, where control plants were kept in the dark for 4 or 8 h at 20 °C. (DOCX 20 kb) [file 11105_2017_1035_MOESM5_ESM.docx]

| Gene expression data of light treated Nure plants 15/20°C | | | | | | | | | | | | | |
| --- | --- | --- | --- | --- | --- | --- | --- | --- | --- | --- | --- | --- | --- |
| **A** |  | CBF14 | PHYA | | PHYB | | PHYC | | CRY1A | | | CRY1B | CRY2 |
| 15 °C 4h/20 °C 4h D | R | 24,52±2,71 | 0,01±0,00 | | 0,01±0,00 | | 0,02±0,00 | | 0,73±0,25 | | | 0,08±0,02 | 0,48±0,07 |
|  | FR | 10,97±1,68 | 0,08±0,01 | | 0,36±0,02 | | 0,13±0,01 | | 0,53±0,04 | | | 0,26±0,01 | 2,07±0,14 |
|  | B | 75,61±2,36 | 0,01±0,00 | | 0,03±0,00 | | 0,02±0,00 | | 0,86±0,19 | | | 0,09±0,00 | 0,32±0,06 |
| 15 °C 8h/20 °C 8h D | R | 25,52±2,96 | 0,00±0,00 | | 0,02±0,00 | | 0,01±0,00 | | 0,76±0,16 | | | 0,14±0,01 | 0,40±0,01 |
|  | FR | 10,37±1,05 | 0,18±0,02 | | 0,36±0,01 | | 0,12±0,01 | | 0,91±0,33 | | | 0,71±0,12 | 1,75±0,09 |
|  | B | 82,75±7,51 | 0,00±0,00 | | 0,07±0,01 | | 0,01±0,00 | | 0,95±0,38 | | | 0,15±0,00 | 0,40±0,04 |
| Gene expression data of light treated Cheyenne plants 15/20°C | | | | | | | | | | | | |  |
| **B** |  | CBF14 | PHYA | | PHYB | | | PHYC | | CRY1A | CRY2 | |  |
| 15 °C 4h/20 °C 4h D | R | 14,29±0,21 | 0,32±0,05 | | 0,42±0,22 | | | 0,33±0,01 | | 2,83±0,23 | 0,67±0,07 | |  |
|  | FR | 9,70±0,38 | 0,64±0,12 | | 0,54±0,15 | | | 0,34±0,04 | | 2,28±0,19 | 2,33±0,32 | |  |
|  | B | 38,15±0,81 | 1,32±0,41 | | 1,39±0,22 | | | 0,92±0,08 | | 4,60±0,29 | 0,59±0,01 | |  |
| 15 °C 8h/20 °C 8h D | R | 4,68±0,21 | 0,25±0,01 | | 1,02±0,04 | | | 0,40±0,02 | | 2,72±0,29 | 0,23±0,02 | |  |
|  | FR | 5,25±0,12 | 0,36±0,03 | | 0,49±0,09 | | | 0,23±0,02 | | 1,41±0,20 | 1,42±0,21 | |  |
|  | B | 35,03±1,11 | 0,28±0,03 | | 1,86±0,53 | | | 0,34±0,03 | | 3,39±0,31 | 0,37±0,04 | |  |
| Gene expression data of light treated G3116 plants 15/20°C | | | | | | | | | | | | |  |
| **C** |  | CBF14 | | PHYA | PHYB | PHYC | | | | CRY1A | | CRY2 |  |
| 15 °C 4h/20 °C 4h D | R | 21,13±1,16 | | 0,83±0,02 | 0,49±0,02 | 0,49±0,06 | | | | 1,20±0,11 | | 0,88±0,11 |  |
|  | FR | 5,00±0,52 | | 0,62±0,01 | 0,39±0,02 | 0,47±0,01 | | | | 0,92±0,14 | | 2,06±0,15 |  |
|  | B | 106,40±0,74 | | 0,66±0,06 | 0,67±0,08 | 0,58±0,05 | | | | 1,65±0,06 | | 1,76±0,27 |  |
| 15 °C 8h/20 °C 8h D | R | 19,30±0,67 | | 0,93±0,03 | 1,33±0,09 | 1,08±0,07 | | | | 2,18±0,09 | | 0,76±0,03 |  |
|  | FR | 12,67±0,09 | | 0,31±0,08 | 0,49±0,06 | 0,14±0,02 | | | | 1,51±0,62 | | 1,48±0,23 |  |
|  | B | 96,38±3,72 | | 0,57±0,06 | 0,87±0,08 | 0,44±0,03 | | | | 1,17±0,06 | | 0,61±0,05 |  |
