# Supplementary figures and images for: Light and Temperature Signalling at the Level of CBF14 Gene Expression in Wheat and Barley
Source: Plant Mol Biol Report. 2017 May 12;35(4):399–408. doi: 10.1007/s11105-017-1035-1 (PMC5504222; doi:10.1007/s11105-017-1035-1)

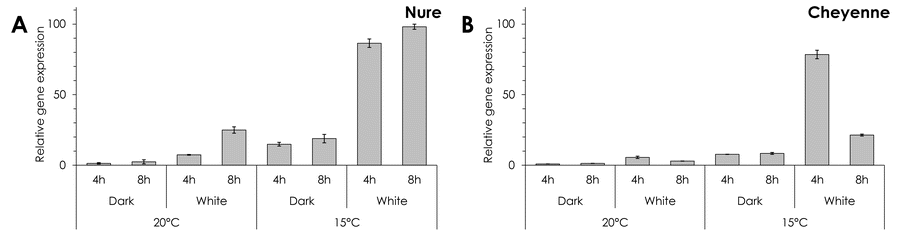

Supplement: Supplementary file 6 — The effect of white light on CBF14 expression. Relative expression of CBF14 at 20 °C or 15 °C after 4 or 8 h of white light treatment in Nure (A), Cheyenne (B). Expression levels presented were calculated using the ΔΔCt method, where Ct values were normalized to the Ct values of house-keeping genes (cyclophilin for panel A and phosphogluconate dehydrogenase for panel B). (GIF 15 kb) [file 11105_2017_1035_Fig7_ESM.gif]

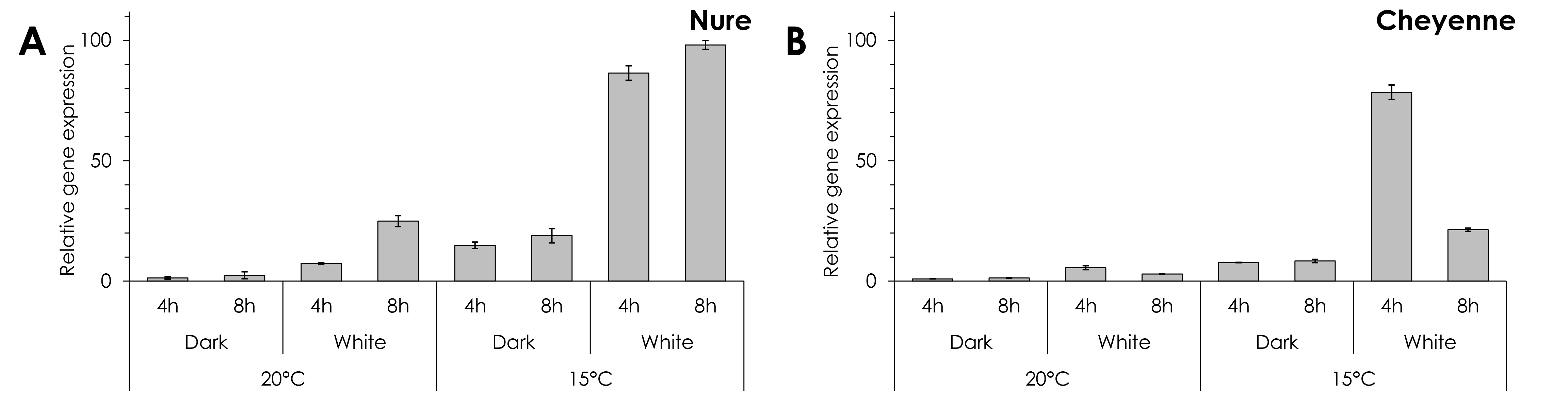

Supplement: Supplementary file 7 — High resolution image (TIFF 480 kb) [file 11105_2017_1035_MOESM6_ESM.tif]

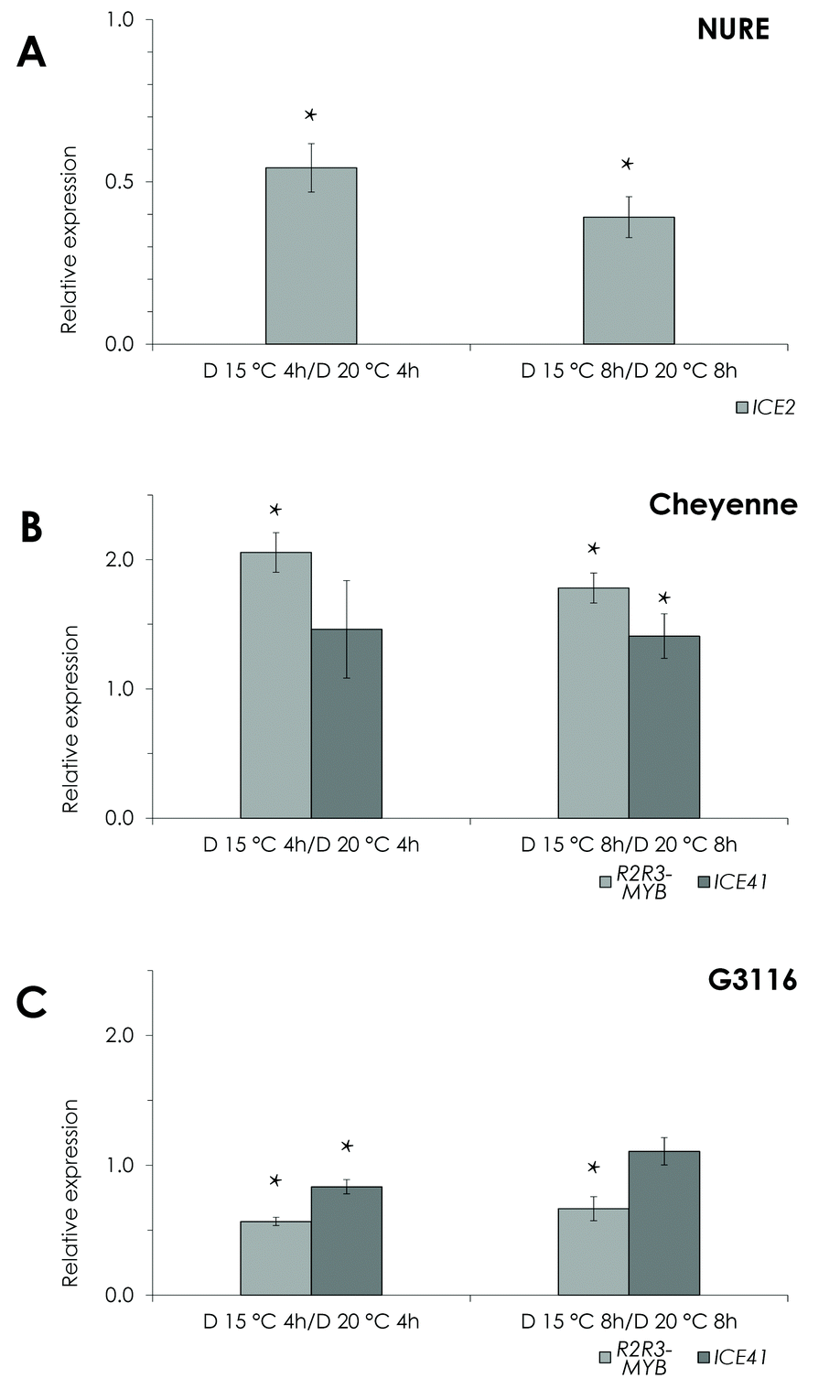

Supplement: Supplementary file 8 — The effect of temperature on ICE2, R2R3-MYB and ICE41 gene expression in the dark. Relative gene expression levels in plants transferred from 20 °C to 15 °C for 4 or 8 h are shown. A) Nure, B) Cheyenne, C) G3116. Expression levels were calculated using the ΔΔCt method and were normalised to the values from the control plants, which were kept at 20 °C for 4 or 8 h. * Significant at the level of P < 0.05 compared with the 4- or 8-h control samples. Results of the comprehensive set of statistical analysis are shown in S2.Table (GIF 99 kb) [file 11105_2017_1035_Fig8_ESM.gif]

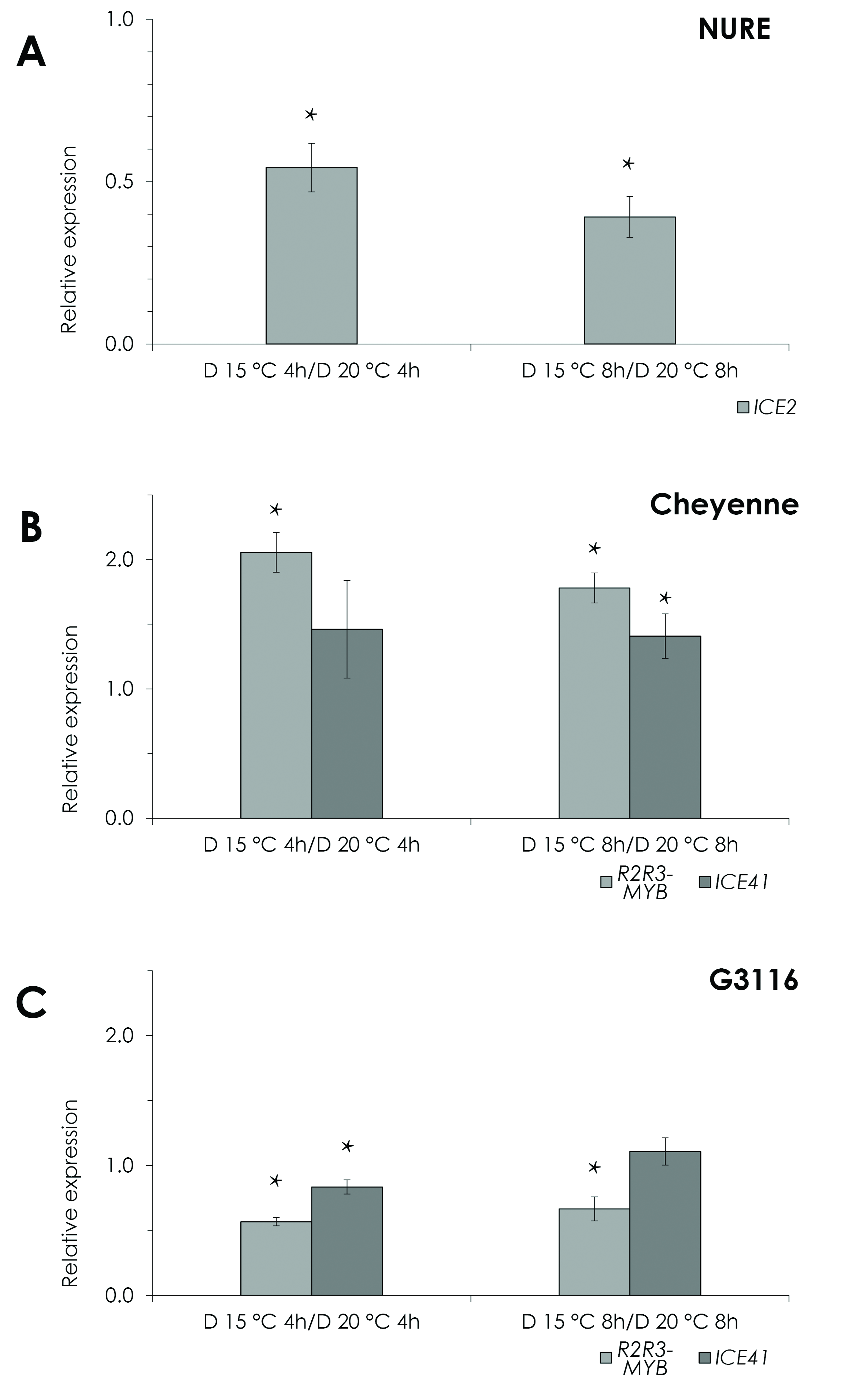

Supplement: Supplementary file 9 — High resolution image (TIFF 1917 kb) [file 11105_2017_1035_MOESM7_ESM.tif]

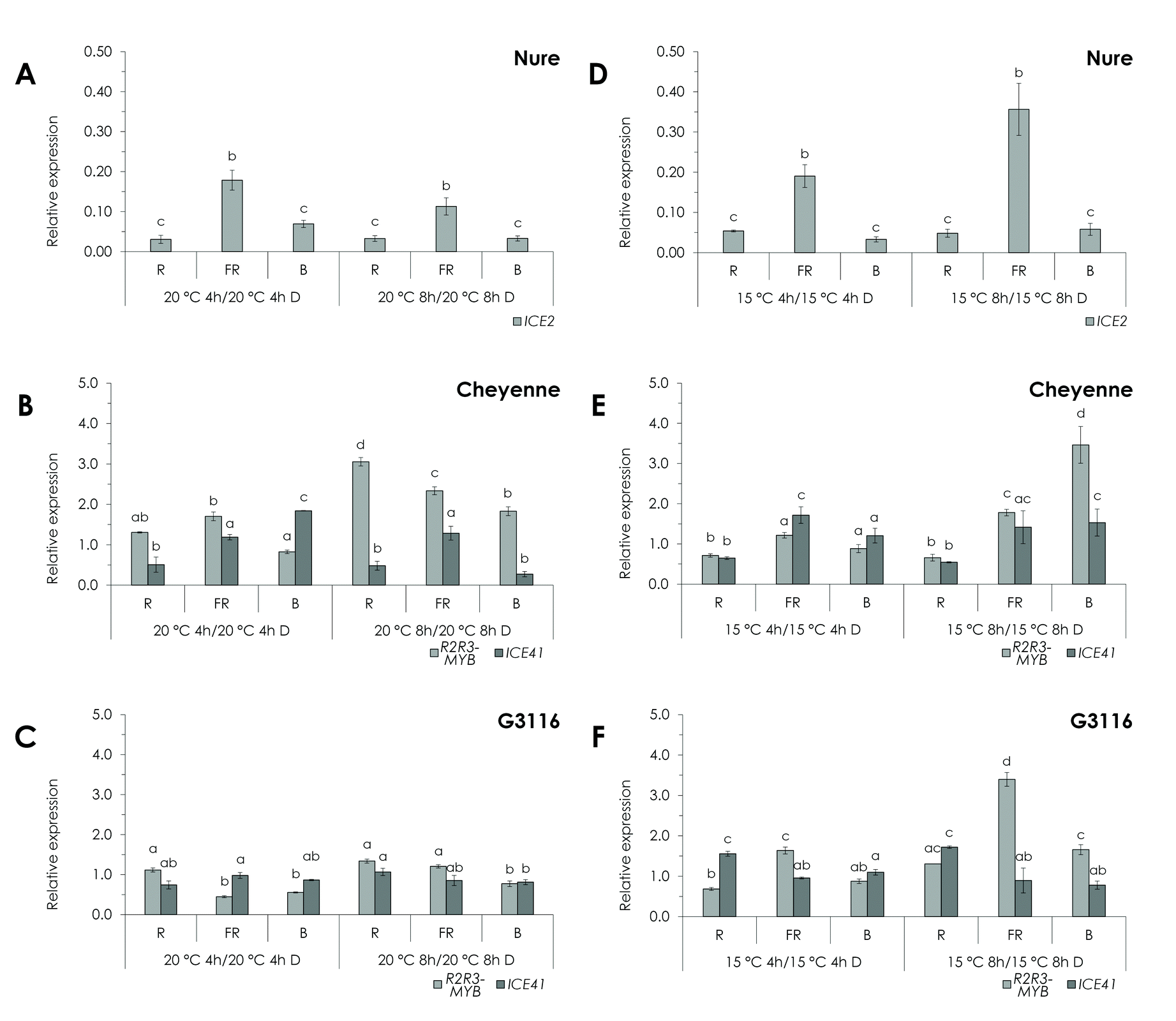

Supplement: Supplementary file 10 — The effect of light on ICE2, R2R3MYB and ICE41 gene expression. A-C) Relative gene expressions at 20 °C after 4 or 8 h of R, FR and B light treatment in Nure (A), Cheyenne (B) and G3116 (C). Control plants were kept in the dark for 4 or 8 h at 20 °C. D-E) Relative expression at 15 °C after 4 or 8 h of R, FR and B light treatment in Nure (D), Cheyenne (E) and G3116 (F). Control plants were kept in the dark for 4 or 8 h at 15 °C. Different letters indicate statistically different (P < 0.05) expression levels, where ‘a’ represents the 4- or 8-h control treatment. Results of the comprehensive set of statistical analysis are shown in S2.Table (GIF 191 kb) [file 11105_2017_1035_Fig9_ESM.gif]

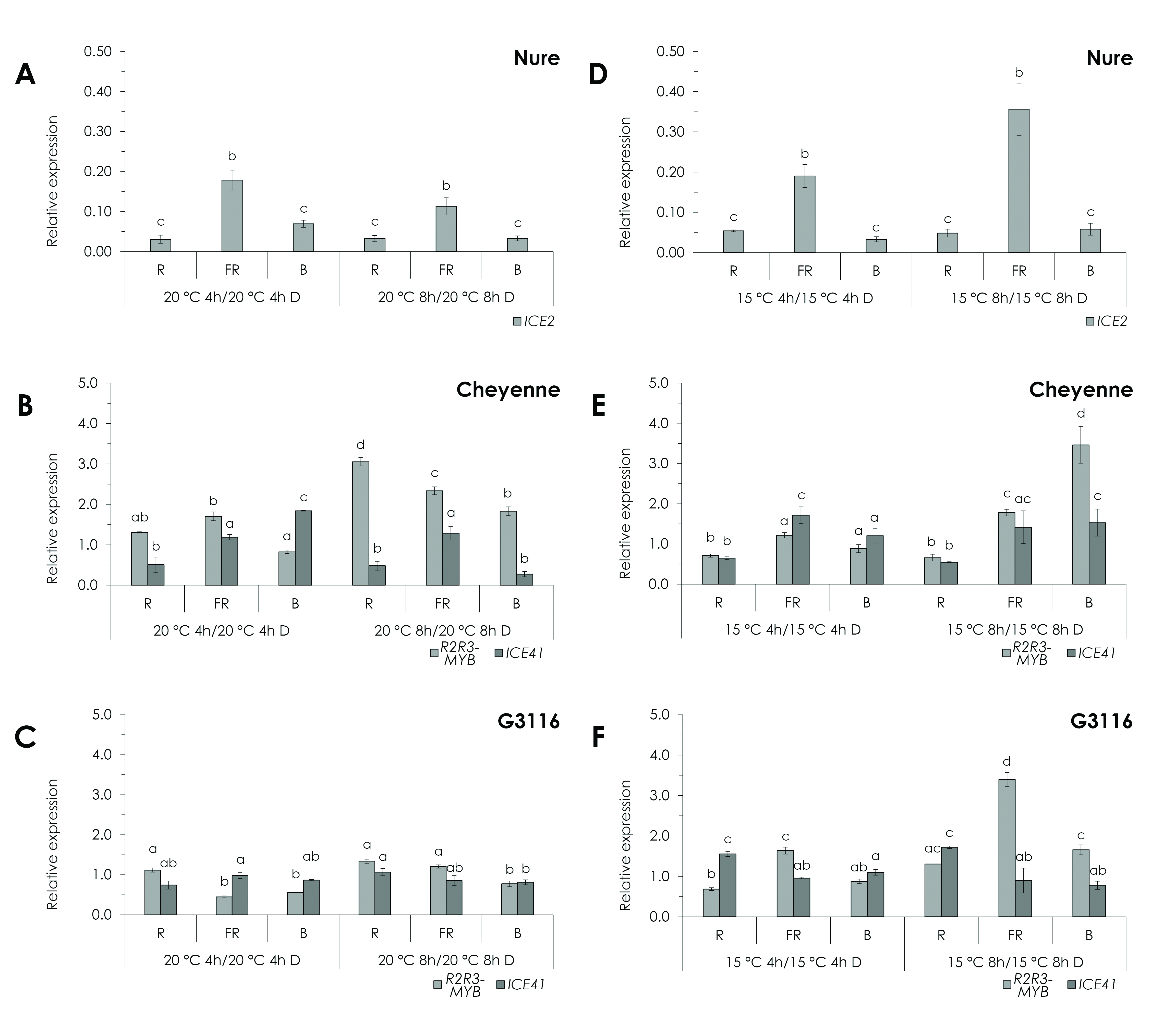

Supplement: Supplementary file 11 — High resolution image (TIFF 2497 kb) [file 11105_2017_1035_MOESM8_ESM.tif]

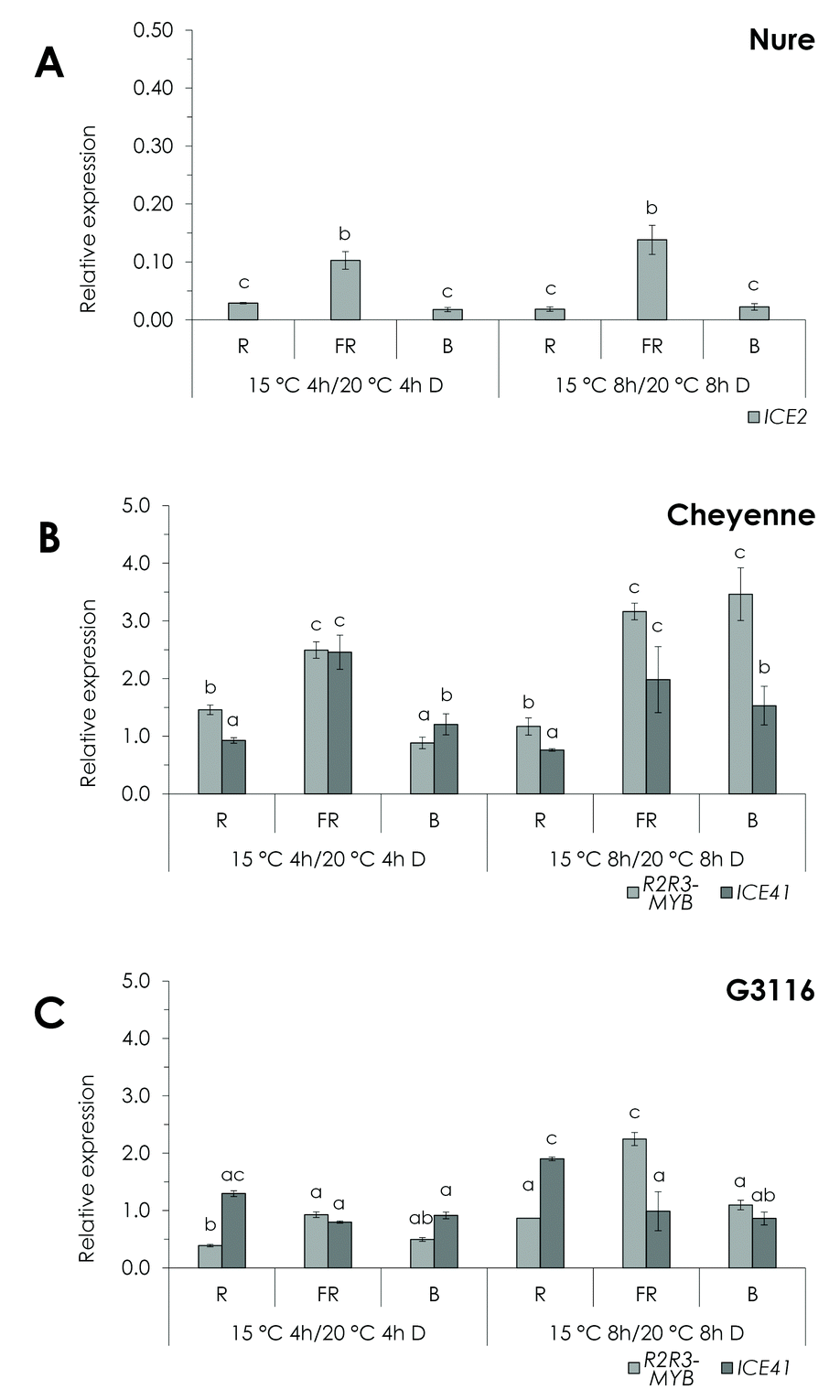

Supplement: Supplementary file 12 — The combined effect of light and temperature on ICE2, R2R3MYB and ICE41 gene expression. Relative expression levels at 15 °C after 4 or 8 h of R, FR and B light treatment in Nure (A), Cheyenne (B) and G3116 (C). Control plants were kept in the dark for 4 or 8 h at 20 °C. Different letters indicate statistically different (P < 0.05) expression levels, where ‘a’ represents the 4- or 8-h control treatment. Results of the comprehensive set of statistical analysis are shown in S2.Table (GIF 93 kb) [file 11105_2017_1035_Fig10_ESM.gif]

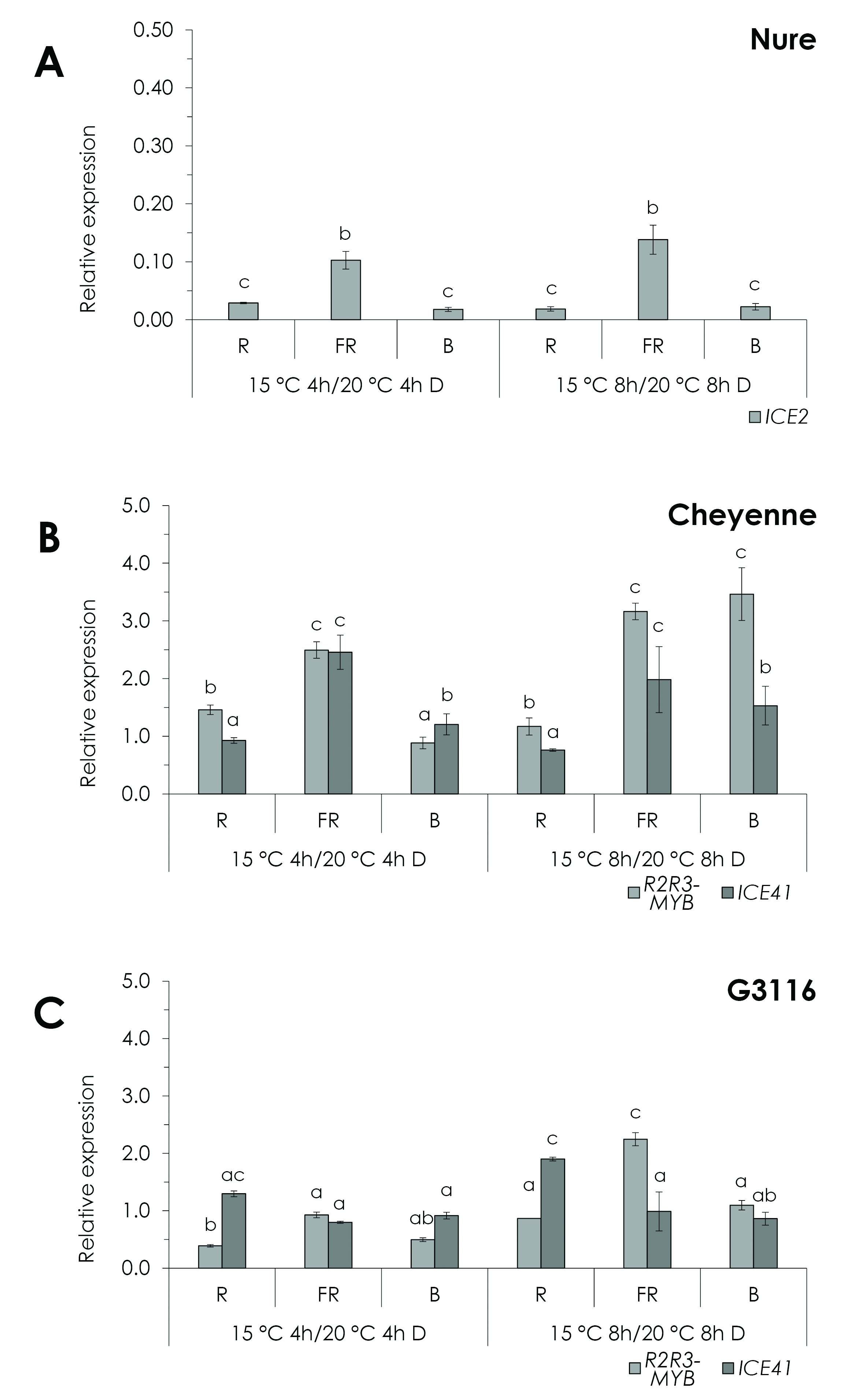

Supplement: Supplementary file 13 — High resolution image (TIFF 1545 kb) [file 11105_2017_1035_MOESM9_ESM.tif]

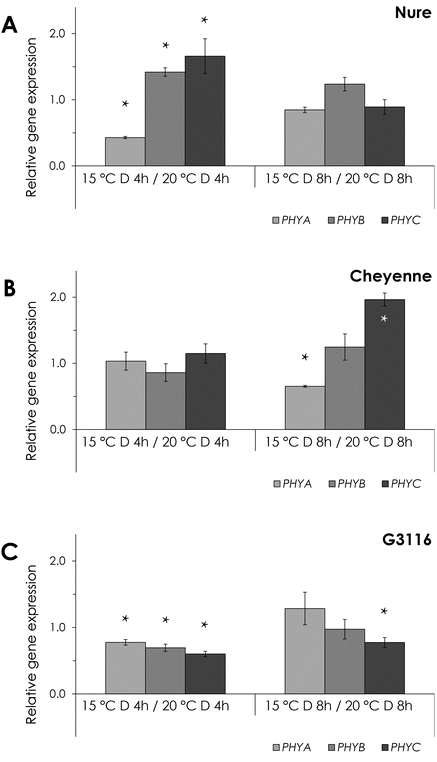

Supplement: Supplementary file 14 — The effect of temperature on phytochrome gene expression in the dark. Relative expression levels of PHYs in plants transferred from 20 °C to 15 °C for 4 or 8 h are shown. A) Nure, B) Cheyenne, C) G3116. Expression levels were calculated using the ΔΔCt method and were normalised to the values from the control plants, which were kept at 20 °C for 4 or 8 h. * Significant at the level of P < 0.05 compared with the 4- or 8-h control samples. (GIF 29 kb) [file 11105_2017_1035_Fig11_ESM.gif]

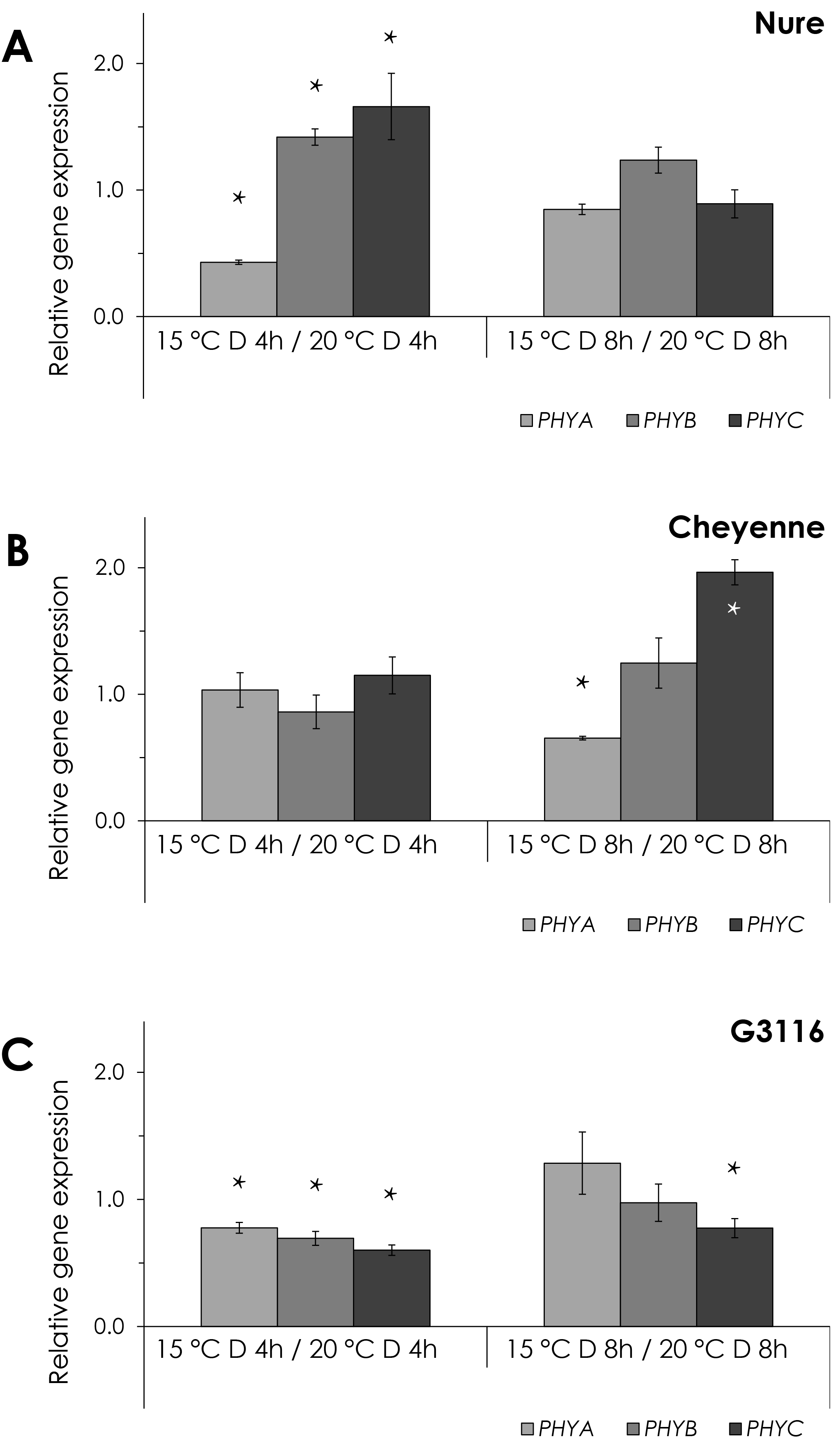

Supplement: Supplementary file 15 — High resolution image (TIFF 1208 kb) [file 11105_2017_1035_MOESM10_ESM.tif]

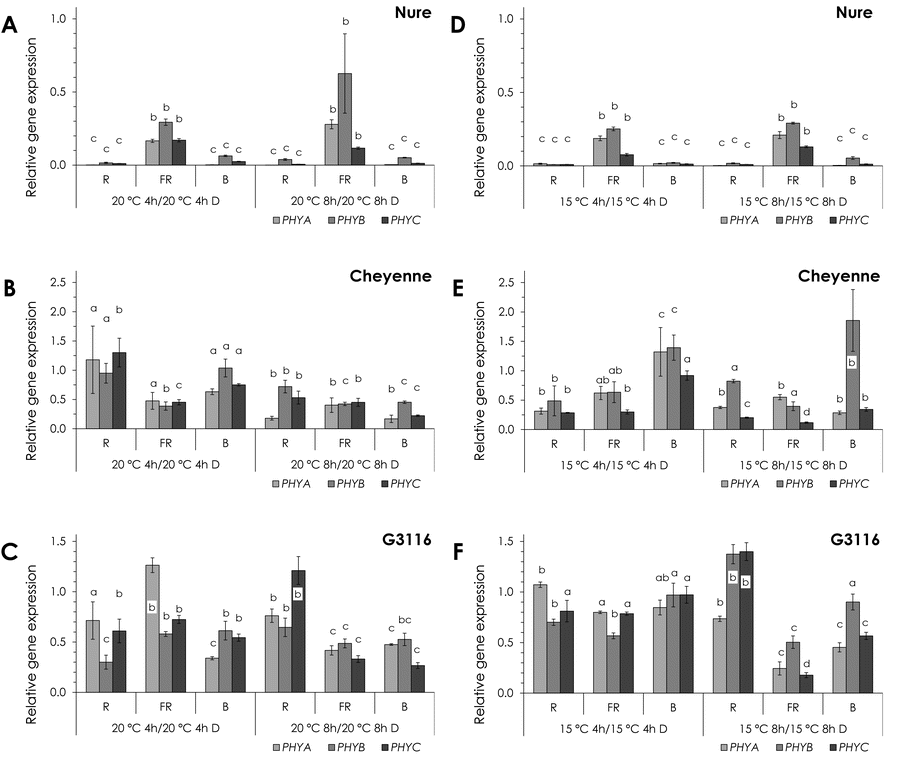

Supplement: Supplementary file 16 — The effect of light on phytochrome gene expression. A-C) Relative expression of PHYs at 20 °C after 4 or 8 h of R, FR and B light treatment in Nure (A), Cheyenne (B) and G3116 (C). Control plants were kept in the dark for 4 or 8 h at 20 °C. D-E) Relative expression of PHYs at 15 °C after 4 or 8 h of R, FR and B light treatment in Nure (D), Cheyenne (E) and G3116 (F). Control plants were kept in the dark for 4 or 8 h at 15 °C. Different letters indicate statistically different (P < 0.05) expression levels, where ‘a’ represents the 4- or 8-h control treatment. (GIF 67 kb) [file 11105_2017_1035_Fig12_ESM.gif]

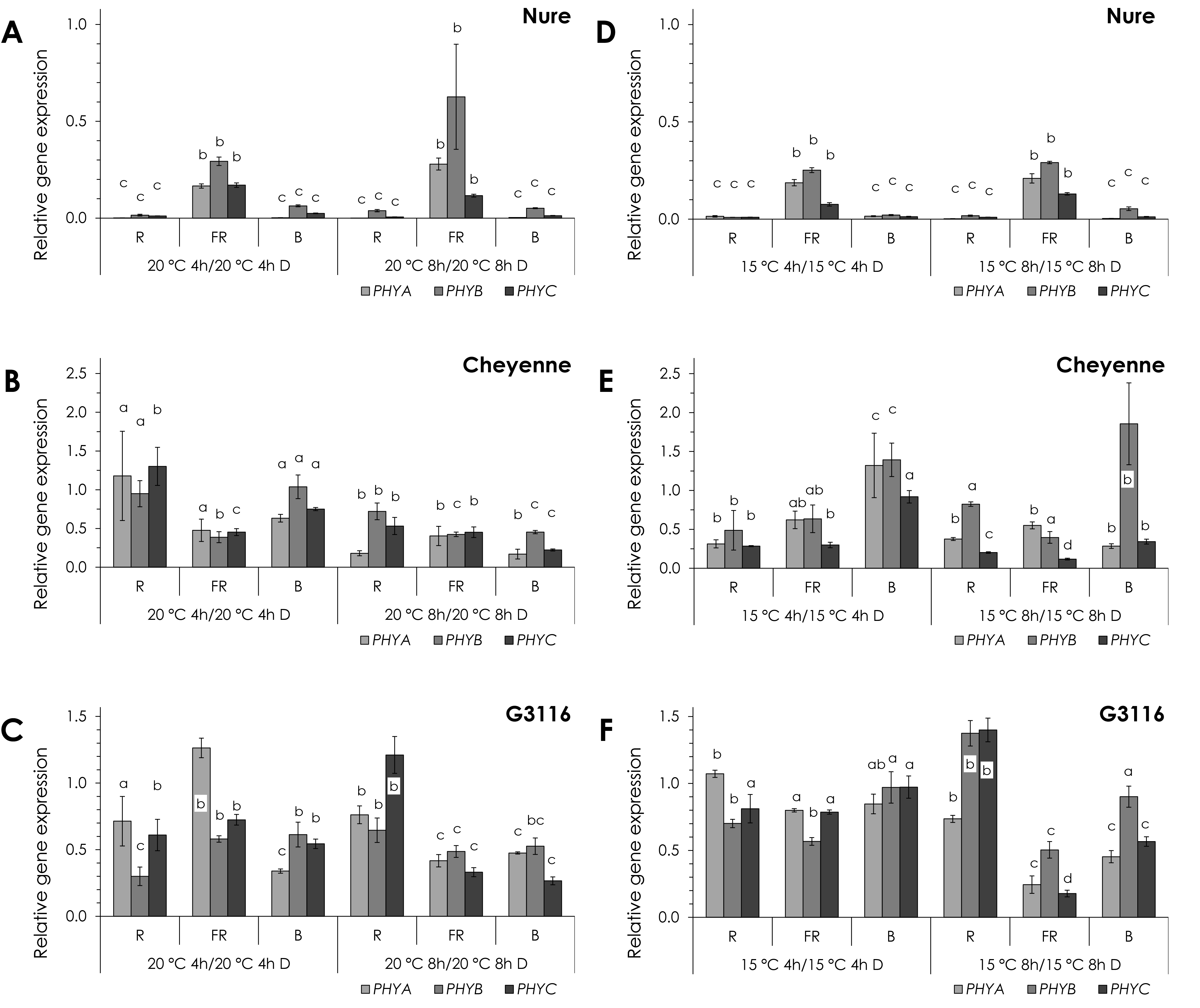

Supplement: Supplementary file 17 — High resolution image (TIFF 1817 kb) [file 11105_2017_1035_MOESM11_ESM.tif]

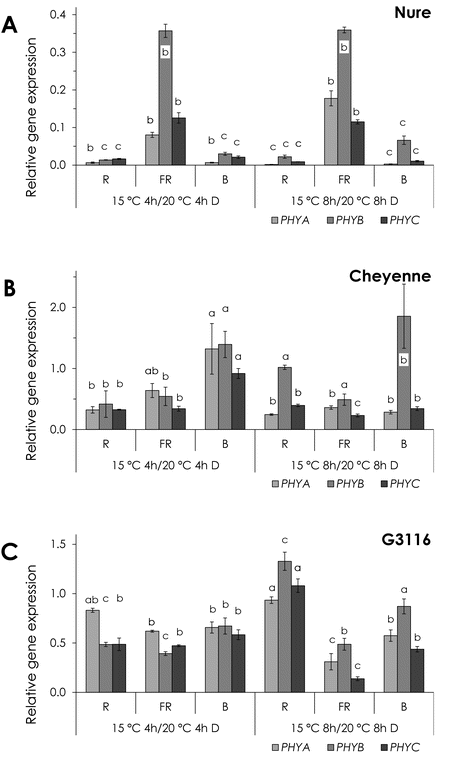

Supplement: Supplementary file 18 — The combined effect of light and temperature on phytochrome gene expression. Relative expression of PHYs at 15 °C after 4 or 8 h of R, FR and B light treatment in Nure (A), Cheyenne (B) and G3116 (C). Control plants were kept in the dark for 4 or 8 h at 20 °C. Different letters indicate statistically different (P < 0.05) expression levels, where ‘a’ represents the 4- or 8-h control treatment. (GIF 35 kb) [file 11105_2017_1035_Fig13_ESM.gif]

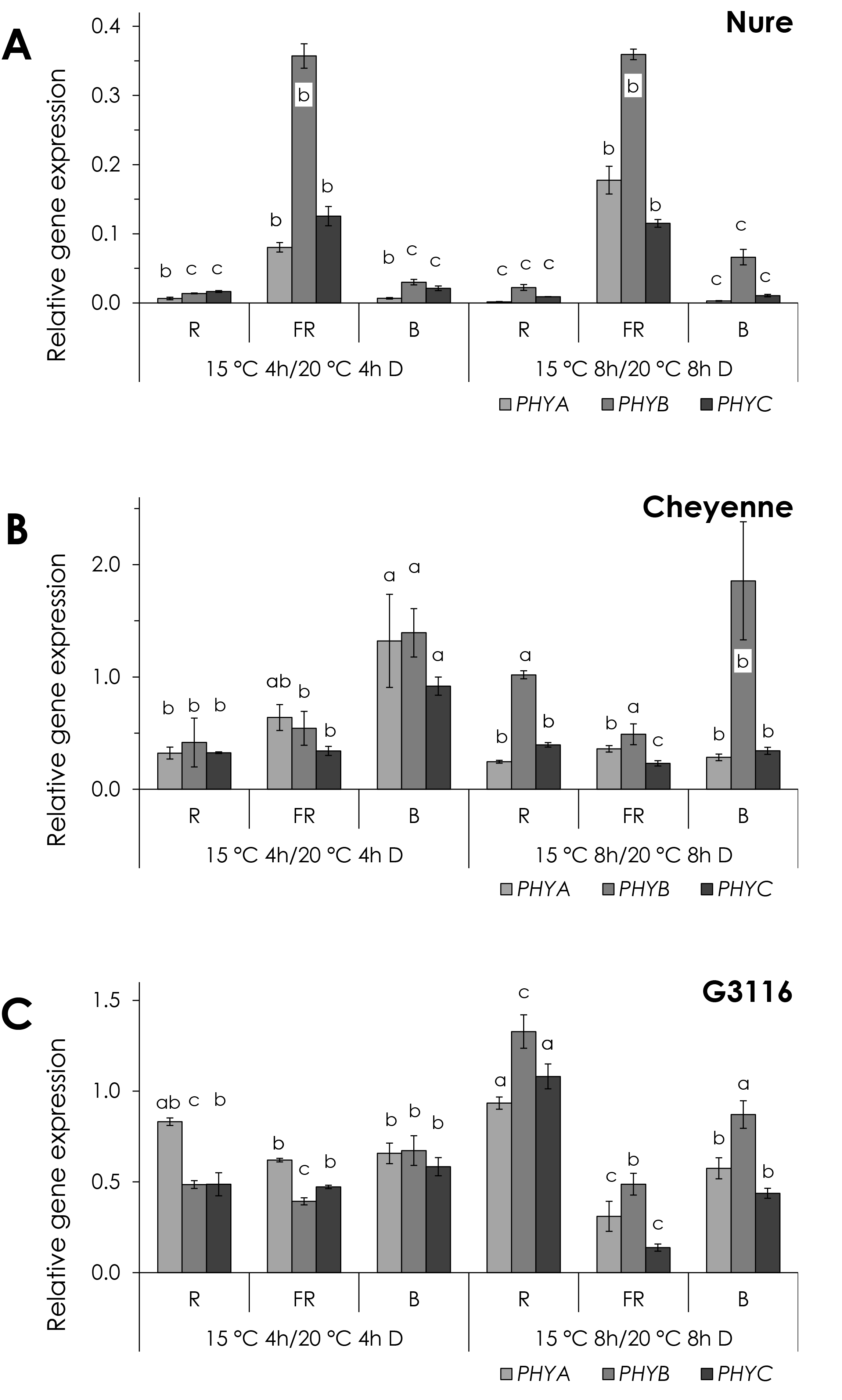

Supplement: Supplementary file 19 — High resolution image (TIFF 1236 kb) [file 11105_2017_1035_MOESM12_ESM.tif]
